# SmMYB44, a transcriptional repressor, negatively regulates anthocyanin biosynthesis in eggplant (*Solanum melongena* L.)

**DOI:** 10.3389/fpls.2026.1927261

**Published:** 2026-10-26

**Authors:** Linzhi Li, Mengliang Zhu, Xin Pang, Jie Yang, Shaohang Li, Qijun Ma, Huoying Chen

**Affiliations:** 1 College of Modern Horticulture, Suzhou Polytechnic Institute of Agriculture, Suzhou, China; 2 School of Agriculture and Biology, Shanghai Jiao Tong University, Shanghai, China

**Keywords:** anthocyanin, eggplant, SmMYB44, transcriptional repressor, SmTPL

## Abstract

Anthocyanins are the main flavonoid secondary metabolites in plants. They not only protect plants against diverse biotic and abiotic stresses but also provide human health benefits. In this study, SmMYB44, an R2R3-MYB transcription factor, was identified from the photosensitive eggplant cultivar ‘Lanshan Hexian’. SmMYB44 was localized in the nucleus, induced by light, and expressed specifically in peel, petals and roots. Overexpression of *SmMYB44* in transgenic eggplant significantly inhibited anthocyanin accumulation, whereas virus-induced gene silencing of *SmMYB44* in fruit peels markedly increased anthocyanin content and upregulated the transcription levels of key biosynthetic genes *SmCHS* and *SmF3H*. Furthermore, Agrobacterium-mediated CRISPR/Cas9 editing of *NtMYB44* in tobacco cultivar K326, with an editing efficiency of 84.62%, markedly suppressed *NtMYB44* transcription and strongly upregulated anthocyanin biosynthetic genes in tobacco petals, resulting in a 2.5- to 4.2-fold increase in anthocyanin content and significantly darker petal color. Yeast one-hybrid and dual-luciferase assays confirmed that SmMYB44 could directly bind to the promoters of *SmCHS* and *SmF3H* and repress their transcriptional activity. In addition, SmMYB44 physically interacts with the co-repressor SmTPL to form a transcriptional repressive complex. Although SmTPL alone has no capacity to activate *SmCHS* and *SmF3H* transcription, it cooperates with SmMYB44 to strengthen the repression of target gene promoters. Collectively, SmMYB44 negatively regulates light-induced anthocyanin biosynthesis in eggplant through direct transcriptional repression of key structural genes and synergistic inhibition through physical association with the co-repressor SmTPL.

## Introduction

Anthocyanin is a water-soluble flavonoid secondary metabolite widely distributed in plants (Liu et al., 2021b). As a natural pigment, it not only imparts rich red, purple, blue, and other colors to plant organs but also helps plants resist various biotic and abiotic stresses, such as ultraviolet radiation, drought, low temperature, and pathogen infection (Kaur et al., 2023). Additionally, due to its strong antioxidant activity, it has multiple health benefits for the human body, making it a key factor in the quality improvement of horticultural crops (Avula et al., 2023; Panda et al., 2025). Furthermore, the biosynthesis and accumulation of anthocyanins in most horticultural crops exhibit typical light dependence (Lasmar Dos Reis et al., 2026). A low-light environment often directly inhibits pigment accumulation, thereby affecting crop commodity value.

To date, the biosynthetic pathway of anthocyanin has been systematically elucidated in Arabidopsis, apple, peach, tomato, and other model plants and horticultural crops (Chen et al., 2026). This pathway is highly conserved and involves a series of enzymatic cascades encoded by key structural genes. The core catalytic enzymes include chalcone synthase (CHS), chalcone isomerase (CHI), flavanone 3-hydroxylase (F3H), flavonoid 3’-5’-hydroxylase (F3’5’H), dihydroflavonol reductase (DFR), anthocyanin synthase (ANS), and glycosyltransferase (UFGT) (Li et al., 2022b). The orderly expression of these genes directly determines the synthesis and accumulation of anthocyanins.

Transcriptional level regulation is the core mechanism governing anthocyanin biosynthesis. In higher plants, the MBW complex, formed by R2R3-MYB, bHLH, and WD40 transcription factors, serves as the core regulatory module for the expression of anthocyanin synthesis structural genes (Li et al., 2022b; Yang et al., 2022) This regulatory model is highly conserved across most species. Within this framework, numerous R2R3-MYB family members function as positive regulators, activating and controlling anthocyanin synthesis. For example, *Arabidopsis thaliana* AtMYB75/PAP1 and AtMYB90/PAP2, tomato SlANT1 and SlAN2, apple MdMYB10 and MdMYB1, and potato StMYB200/StMYB210 are typical anthocyanin synthesis activators that drive tissue-specific pigmentation in their respective species (Zuluaga et al., 2008; Liu et al., 2021a; Du et al., 2025). AtMYB4 is the first identified R2R3-type anthocyanin negative regulator in *Arabidopsis thaliana*, which directly inhibits the expression of anthocyanin biosynthetic structural genes (Banerjee et al., 2024). Subsequently, an array of R2R3-MYB repressors have been functionally verified, including strawberry FaMYB1, potato StMYB44, and apple MdMYB44 (Aharoni et al., 2001; Liu et al., 2019; Huang et al., 2024). Additionally, R3-MYB factors containing truncated R2 domains, such as Arabidopsis AtMYBL2, AtCPC, and tomato SlMYBATV, can also inhibit MBW complex formation by interfering with its assembly, thereby further expanding the negative regulatory network of the flavonoid pathway (Yan et al., 2020; Xing et al., 2024). These negative regulators accurately control anthocyanin accumulation by antagonizing the positive regulation of the MBW complex, competitively binding to bHLH-interacting proteins, or directly binding to structural gene promoters to inhibit transcription, thereby preventing excessive pigment synthesis.

In recent years, the transcriptional regulation of anthocyanin synthesis in eggplant has been progressively elucidated (Ai et al., 2024). Beyond the classical MBW complex, multiple specific regulatory factors have been identified. Existing studies have confirmed that SmMYB1 (SmMYB113), SmMYB5, and SmMYB6.2 are key positive regulatory MYB factors for anthocyanin synthesis in eggplant. Among these, SmMYB113 serves as the core positive activator of eggplant peel pigmentation, directly binding to the promoters of downstream structural genes to drive anthocyanin accumulation (Jiang et al., 2016). SmMYB5 integrates jasmonic acid and light signals, forms a protein complex with SmTT8, activates the expression of *SmCHS*, *SmF3H* and *SmANS*, and effectively mitigates poor pigmentation under low-light conditions (Li et al., 2024). SmMYB6.2 activates the expression of *SmANS* and coordinates with SmbHLH79 and SmGL3 to regulate anthocyanin biosynthesis in eggplant (Hao et al., 2026). Additionally, light signal-related transcription factors such as SmERF118 participate in anthocyanin regulation, and the SmHY5-induced SmERF118-SmMYB1 complex promotes light-dependent anthocyanin accumulation in eggplant (Li et al., 2025). However, the molecular mechanisms underlying the negative regulation of photosensitive eggplant germplasm remain rarely reported. Recent genome-wide and transcriptome studies have identified SmMYB86 and SmBICs, which directly repress early biosynthetic genes or interfere with MBW complex assembly to fine-tune anthocyanin accumulation (He et al., 2021; Li et al., 2021). Homologs of MYB44 from other species, such as IbMYB44 in sweet potato and PsMYB44 in tree peony, have been verified as negative regulators that reduce anthocyanin content by inhibiting key structural genes or disrupting activator complexes (Wei et al., 2020; Luan et al., 2023). These findings strongly suggest that MYB44-type repressors represent a conserved functional clade in the light-responsive anthocyanin regulatory network across higher plants.

Light is the primary environmental factor regulating plant anthocyanin synthesis (Zhang et al., 2026). It activates downstream light signal transduction pathways by engaging photoreceptors such as HY5 and PIF, thereby precisely regulating the spatiotemporal expression of R2R3-MYB family genes (Qin et al., 2022; Anyatama et al., 2025). The MYB regulatory factors directly bind to cis-acting elements in target gene promoters or interact with proteins antagonistically to achieve bidirectional precise regulation of anthocyanin accumulation, forming a rigorous light-responsive molecular regulatory network (Yan et al., 2023). Previous studies have demonstrated that anthocyanin synthesis in the photoperiod-sensitive eggplant variety ‘Lanshan Hexian’ is strictly light-induced, resulting in nearly white skin following bagging and shading treatments (Li et al., 2017). Upon removal of the bag and exposure to light, anthocyanin content increases rapidly, making this variety an ideal model for studying the regulation of light-responsive anthocyanin synthesis.

In recent years, CRISPR/Cas9 gene editing technology has emerged as a core frontier method for gene function verification and quality improvement in horticultural crops (Xu et al., 2020; Zhang et al., 2024). In species such as tomato, potato, and apple, precise editing of MYB suppressor genes has effectively relieved the negative regulation of anthocyanin synthesis, significantly enhancing fruit pigment accumulation and commercial value (Liu et al., 2022; Mackon et al., 2023). Research on gene editing of MYB negative regulatory factors in eggplant remains unexplored. To investigate the key regulatory genes of light-induced anthocyanin synthesis in this variety, our group conducted transcriptome sequencing analysis under shading and light treatments in the early stages, screening for the differentially expressed gene *SmMYB44*. Transient expression experiments in tobacco preliminarily confirmed its ability to inhibit anthocyanin synthesis; however, its specific molecular regulatory mechanism and the application potential of targeting this gene via gene editing have yet to be elucidated (Li et al., 2017).

Based on the above research background, the full-length coding sequence of *SmMYB44* was cloned from the photosensitive eggplant variety ‘Lanshan Hexian’. Its tissue-specific expression as well as light-inducible transcriptional patterns following fruit bag removal were systematically characterized. Using our previously generated transcriptomic datasets of eggplant peel under shading and re-lighting treatments, co-expression analysis of differentially expressed genes was performed to screen candidate interactors whose expression profiles tightly correlated with *SmMYB44*. Among these candidates, SmTPL, a member of the TOPLESS co-repressor family, exhibited highly coordinated transcriptional dynamics with *SmMYB44*. We further conducted cross-species protein–protein interaction prediction via AraPPINet (http://netbio.sjtu.edu.cn/arappinet/). The prediction results suggested a strong potential physical interaction between the Arabidopsis ortholog of SmMYB44 and AtTPL. Consistent with established knowledge that EAR-motif-containing R2R3-MYB transcriptional repressors generally recruit TPL/TPR co-repressors to assemble functional repressive complexes, SmTPL was prioritized as the core candidate interacting partner of SmMYB44 for subsequent experimental validation. To dissect the biological function of *SmMYB44* in eggplant anthocyanin biosynthesis, three complementary genetic strategies were deployed: Agrobacterium-mediated stable overexpression, virus-induced gene silencing (VIGS), and CRISPR/Cas9 gene editing. Additionally, through yeast one-hybrid assays, dual luciferase reporter gene assays, and protein interaction experiments, the regulatory relationship between *SmMYB44* and downstream key structural genes as well as its interaction protein SmTPL was explored. Consequently, the molecular mechanism by which SmMYB44 negatively regulates anthocyanin synthesis in eggplant was clarified. The objective of this study is to enhance the transcriptional regulatory network of eggplant light-responsive anthocyanin synthesis and to provide high-quality targets and theoretical support for eggplant fruit color improvement and stress-resistant molecular breeding.

## Materials and methods

### Plant materials and growth conditions

The eggplant cultivar *‘*Lanshan Hexian*’, ‘*116*’*and *SmMYB44-OE* transgenic seeds were soaked with 600 mg/L GA overnight to break seed dormancy, followed by disinfection. They were then germinated on sterile filter paper in culture dishes. After 4 days of germination, seedlings were transplanted into plastic pots (5 cm in diameter) filled with a mixture of perlite, vermiculite, and humus in a volume ratio of 1:1:3. These pots were placed in a climate-controlled chamber maintained at day/night temperatures of 25 °C/22 °C, under a 16-h light/8-h dark photoperiod with a light intensity of 200 µmol m^-^² s^-^¹.Seedlings at the four-leaf stage were transplanted to the horticultural experimental farm of Suzhou Polytechnic Institute of Agriculture, China.

The *Nicotiana tabacum* cv. *K326* (pink-flowered tobacco) was selected as the receptor material for stable genetic transformation of the *SmMYB44*-edited.Seed disinfection was performed by immersing seeds in 75% ethanol for 30 s, followed by rinsing with sterile water for 1 minute. Seeds were then disinfected with 84 disinfectants for 3–5 min and rinsed three times with sterile water. Sterilized seeds were inoculated onto MS basal germination medium containing 30 g/L sucrose, 8 g/L agar, and adjusted to a pH of 5.8. The seeds were cultured at 23 °C under a 16-hour light/8-hour dark photoperiod for 4–5 weeks. Aseptic seedlings with uniform growth were selected, and their leaves were used as explants. The T1 generation homozygous editing line and wild-type tobacco plants were transplanted into a solar greenhouse maintained at 25 °C with a 12-hour light/12-hour dark cycle. After 8–10 weeks of natural growth, petal coloration phenotypes of the tobacco plants were observed and photographed at the full-bloom stage.

The seeds of *Nicotiana benthamiana* were sown in plastic pots (10 cm in diameter) and were cultured in growth chamber with a 16-hour light/8-hour dark photoperiod and day/night temperatures of 25 °C/22 °C.

### Isolation of *SmMYB44*

The total RNA was extracted from the eggplant fruit peel, using the Mini BEST Plant RNA Extraction Kit (TaKaRa, Japan) and following the manufacturer’s instructions. Coding sequence of *SmMYB44* (Sme2.5_24183.1_g00002.1) was isolated from the photosensitive eggplant cultivar, ‘Lanshan Hexian’, according to the Eggplant Genome DataBase (http://eggplant.kazusa.or.jp/keyword.html). The specific primers were designed by primer 5 and were listed in **Supplementary Table S3**. The PCR products were fused to pEasy-Blunt Simple Vector (Transgen, China) and were sequenced by the Sangon Biotech (Shanghai, China) to obtain the CDS of *SmMYB44* gene.

### Isolation of *SmTPL*

The full-length CDS of *SmTPL* (Sme2.5_01513.1_g00008.1) was isolated from fruit peel of *‘*Lanshan Hexian*’* following identical RNA extraction, primer design, PCR amplification, T-vector ligation and sequencing procedures described above for SmMYB44. The specific primers were designed by primer 5 and were listed in **Supplementary Table S3**.

### Analysis of subcellular localization

For subcellular localization assays, the PHB-SmMYB44-YFP recombinant vector was constructed using the homologous recombination method, with specific primers provided in **Supplementary Table S3**. *Agrobacterium tumefaciens* strain GV3101 harboring either the PHB-SmMYB44-YFP or the empty PHB-YFP vector was infiltrated into the leaves of 5-week-old *Nicotiana benthamiana* leaves. After infiltration, the *Nicotiana benthamiana* plants were cultured under low-light conditions for 48 h, the epidermal cells of the infiltrated leaves were stained with DAPI (Sangon Biotech, China) to label the nucleus, and fluorescence signals were observed using a laser scanning confocal microscope (Leica TCS SP5-II, Germany).

### Reverse transcription-quantitative real-time PCR

RT-qPCR was performed to analyze the expression patterns of *SmMYB44* and key anthocyanin biosynthesis genes in the following samples: peel tissues of *‘*Lanshan Hexian*’* and *SmMYB-OE* transgenic lines; peel tissues of *TRV2-empty vector-infiltrated* (control) and *SmMYB44-silenced eggplants*; petals of *Nicotiana tabacum* cv. *K326* and T1 homozygous edited lines (derived from selfing of T1 double allelic mutant lines); and root, stem, leaf, petal, sepal, peel, and flesh tissues of *‘*Lanshan Hexian*’* (for tissue-specific expression analysis). For the fruit bagging experiment, uniform eggplant fruits at full bloom were selected and individually covered with light-impermeable paper bags to ensure complete shading. After 14 days of shading, the bags were removed to allow light exposure. Fruit peel were sampled at 0 d, 5 d, and 12 d after bag removal with three biological replicates per time point. Transcriptome data for these time points were obtained from our previous studies (Li et al., 2017). All collected plant materials were immediately frozen in liquid nitrogen and stored at -80 °C, three independent biological replicates per sample. Total RNA was treated with DNase to remove traces of DNA. cDNA synthesis from 1 μg RNA and RT-qPCR were performed as described previously (Li et al., 2021), using PrimeScript RT Master Mix Perfect Real Time Kit (TaKaRa, Japan) and SYBR Premix Ex Taq II Kit (TaKaRa, Japan) on Light Cycler 96 (Roche, Canada), respectively. The RT-qPCR program was: 95 °C for 30 s, followed by 40 cycles of 95 °C for 5 s, 60 °C for 30 s, and 72 °C for 30 s. Each sample was analyzed with three technical replicates using gene-specific primers (**Supplementary Table S3**). *Nicotiana tabacum Actin7* (LOC107831145) and eggplant *Actin* (GU984779.1) were used as internal reference genes. Relative gene expression analyzed using the 2^-ΔΔCT^ method. The transcriptome data from our previous studies (Li et al., 2017).

### Vector construction and transformation of eggplant

The coding sequence of *SmMYB44* was PCR-amplified and cloned into the CaMV35S-promoter-driven PHB vector to generate the 35S::*SmMYB44* construct. This construct was verified by PCR and Sanger sequencing prior to Agrobacterium-mediated transformation into eggplant *‘Lanshan Hexian’*, following the protocol reported previously (Li et al., 2021; Li et al., 2022a). For *SmMYB44* silencing, a 300-bp specific fragment predicted by the SGN VIGS Tool was cloned into the TRV2 vector to generate *TRV2-SmMYB44*, which was also verified by PCR and sequencing. Agrobacterium GV3101 harboring TRV1, empty TRV2, or *TRV2-SmMYB44* was cultured to the logarithmic phase, resuspended in infiltration buffer (10 mM MgCl_2_, 10 mM MES, 200 μM acetosyringone, pH 5.6) to an OD_600_ of 1.0, then mixed with TRV1 at a 1:1 volume ratio. The mixture was dark-incubated for 2–3 h and subsequently infiltrated into the pre-bagged peels of eggplant fruits. Calyxes were covered using opaque paper bags during the full-flowering period, fruits were harvested 14 days after opening the bags. The infiltrated fruits were first dark-cultured for 24 h, followed by low-light culture for 5 days. Fruit peel samples were collected immediately, frozen in liquid nitrogen, and stored at -80 °C for anthocyanin determination. The VIGS experiment was performed with minor modifications based on the previously reported method. To ensure phenotypic consistency, all eggplant fruits were harvested and photographed at an identical developmental stage, specifically 18 days post-anthesis.

### Vector construction and CRISPR/Cas9 editing of tobacco (K326)

Based on the CDS sequence of *SmMYB44*, the tobacco endogenous gene LOC107767835 (*NtMYB44*) was identified as the tobacco-native *MYB44*-like ortholog of eggplant *SmMYB44.* target sites were designed using the online tool available at http://crispr.hzau.edu.cn/CRISPR2/. The target site was synthesized via gene synthesis and cloned into the pUC57 vector, which served as the plasmid template. A 50 μL PCR reaction system was prepared using 1 μL of template, 2 μL each of upstream and downstream primers for the target unit, 25 μL of Biorun Pfu PCR Mix, and 20 μL of water. The PCR program was set as follows: 94 °C for 5 min, followed by 30 cycles of 94 °C for 30 s, 55 °C for 45 s, 72 °C for 23 s and 72 °C for 10 min, 16 °C for 30 min to amplify the target site. Subsequently, 4 μL of the target fragment, 1 μL of T4 DNA ligase, 2 μL of T4 buffer, 1 μL of the restriction enzyme BsaI/EcoRI, 4 μL of CRISPR Cas9 editing vector, and 8 μL of water were mixed to prepare a 20 μL ligation reaction. The ligation and digestion were performed with the following program: 37 °C for 20 min, (37 °C for 10 min, 20 °C for 5 min) for 5 cycles, 37 °C for 1 min and 80 °C for 5 min. The target site was thus inserted into the editing vector CRISPR Cas9 editing vector through restriction-ligation cloning.

Add 1 µL carrier plasmid into 50 µL Agrobacterium GV3101 receptive cells, transferred into electroporation cuvette, add 1 mL LB liquid medium after electroporation, mix well and absorb into 1.5 mL centrifuge tube, and oscillate and culture at 180rpm at 30 °C in a shaker for 30 min. The activated agrobacterium solution was absorbed 50 µL and inoculated on LB solid medium and incubated in the dark at 30 °C for 48 h. Subsequently, single colonies were picked to prepare a reaction system of 20 μL (1μL each of F/R primers, 10 μL of 2×Taq PCR Mix, 1μL of template, and 7 μL of water) for PCR testing of positive clones. Tobacco seeds are disinfected with 75% alcohol for 30 s, washed with sterile water for 1 min, then disinfected with 84 disinfectants for 3 to 5 min, and washed with sterile water three times, 1 min each time. The disinfected tobacco seeds were sown on the germination medium and cultured at 23 °C for 16h/8h under light/in the dark for 4–5 weeks. The sterile tobacco leaves were cut into small pieces with a scalpel and inoculated on the pre-culture medium. Pick Agrobacterium into the infection solution to prepare Agrobacterium resuspension with OD_600_ = 0.2. Inoculate tobacco leaves that have been pre-cultured for 2–3 days into the Agrobacterium suspension for infection for 10–15 min. Inoculate the infected tobacco leaves onto filter paper, dry them, and then inoculate them onto the co-culture medium for dark culture for 48–72 h. The leaves after co-culture for 2 days were transferred to the induction medium to induce callus for about 10 days until callus tissue grew. Select the eligible callus tissues, inoculated them onto the hygromycin resistance screening medium, and cultured them at 23 ± 2 °C for 15–30 days. Inoculate the vigorously growing positive callus from the second screening onto the differentiation medium, 4–5 callus per dish and incubate at 23 °C for 16 h/8 h under light/in the dark for 15–30 days. During the differentiation process, if callus forms seedlings, they should be inoculated onto the medium for strong seedlings and grow for 7 to 10 days. The genomic DNA of positive seedlings was extracted by the CTAB method and the positive seedlings were detected by PCR. Subsequently, design specific detection primers based on the target. After PCR amplification, first-generation sequencing was performed to determine the editing mutations.

### Extraction and quantification of anthocyanins

100 mg of fresh samples were used for anthocyanin isolation and quantitative detection following the previously reported protocol from Jiang et al (Jiang et al., 2016). The total anthocyanin content was calculated using the formula: TA = A × MW × 5 × 100 × V/e, where TA represents the total anthocyanin content (mg/100 g), V indicates the final extraction volume (mL), and A is the absorbance value defined as A = [A510 nm (pH 1.0) − A700 nm (pH 1.0)] − [A510 nm (pH 4.5) − A700 nm (pH 4.5)]. All experiments were performed with no fewer than three independent biological replicates, and each sample was measured in technical triplicate.

### Yeast one-hybrid assay

The DNA-binding capabilities of the SmMYB44 and SmTPL proteins were investigated using a yeast one-hybrid system. The coding sequences (CDS) of *SmMYB44* and *SmTPL* were cloned individually into the pB42AD vector to construct the effector plasmids pB42AD-SmMYB44 and pB42AD-SmTPL, respectively. Promoters of *SmCHS* (Sme2.5_13923.1_g00001.1) *and SmF3H* (Sme2.5_00015.1_g00020.1) were inserted into the LacZ vector to generate the reporter plasmids LacZ-*SmCHS a*nd LacZ-*SmF3H*, respectively. Recombinant effector and reporter plasmids were co-transformed into the yeast strain EGY48a to detect DNA-protein interactions. pB42AD-SmMYB44, pB42AD-SmTPL and LacZ, pB42AD and LacZ-*SmCHS*, and LacZ-*SmF3H* were co-transformed as negative controls. Transformants were initially cultured on SD/-Trp/-Ura medium, and positive interactions were identified by their growth on SD/-Trp/-Ura medium supplemented with X-gal, as determined by colorimetric detection.

### Dual-luciferase assay

The pGreen 0800-LUC vector was used to analyze *in vivo* interactions between transcription factors (TFs) and their target promoters as previously described with minor modifications (Li et al., 2021). Briefly, the coding sequences (CDS) of *SmMYB44* and *SmTPL* were cloned into the PHB vector to construct effector plasmids, with the empty PHB vector serving as a negative control. The promoter sequences of *SmCHS* and *SmF3H* were inserted into the pGreen 0800-LUC vector to generate reporter plasmids. These reporter plasmids were then co-transformed with the helper plasmid pSoup19 into *Agrobacterium tumefaciens* strain GV3101.The effector and reporter strains were then cultured and re-suspended with infiltration buffer (10 mM MES, 10 mM MgCl_2_, 100 µM AS, pH 5.6) to an OD_600_ of 1.0-1.5. The effector and reporter bacterial suspensions were mixed at a volume ratio of 2:1 and then infiltrated into the leaves of 6-leaf-stage *Nicotiana benthamiana* plants using needleless syringes. Three days post-infiltration, firefly luciferase (LUC) and Renilla luciferase (REN) activities in infiltrated leaves were quantified using the Dual Luciferase Reporter Assay System (Promega, USA) according to the manufacturer’s protocol. The trans-repression activity of SmMYB44 and SmTPL to target promoters was determined by the LUC/REN ratio. At least six biological replicates were tested for each combination.

### Yeast two-hybrid assay

The *SmMYB44* coding sequence was cloned into pGBKT7 (BD) as bait, and the *SmTPL* CDS was inserted into pGADT7 (AD) as prey. The recombinant bait and prey plasmids were co-transformed into the yeast strain AH109 via the lithium acetate-mediated transformation method, following the manufacturer’s protocol (Clontech, USA). Transformed yeast were cultured on SD/-Trp-Leu medium at 30 °C for 3 d. Six colonies were then inoculated on SD/-Trp-Leu-His and SD/-Trp-Leu-His-Ade media and incubated at 30 °C for 3 d to detect protein interactions.

### Bimolecular fluorescence complementation

For BiFC analysis, the vectors pXY104 and pXY106 were described previously (Li et al., 2021; Li et al., 2022a). The coding sequence of *SmMYB44* and CDS of *SmTPL* were amplified via PCR and cloned, respectively, into the pXY104 and pXY106 vectors to generate SmMYB44-cYFP and SmTPL-nYFP fusion constructs. The validated plasmids were transformed into *Agrobacterium tumefaciens* strain GV3101 via heat shock. Positive transformants were cultured overnight, harvested by centrifugation, and resuspended in infiltration buffer to an OD_600_ of 0.5. The two bacterial suspensions were mixed equally, pre-incubated, and co-infiltrated into the abaxial leaves of 4-6-week-old *Nicotiana benthamiana* plants. After 48 h of incubation under a 16-h light/8-h dark cycle at 25 °C, YFP fluorescence was visualized using a Leica TCS SP5-II confocal microscope, with empty vectors serving as negative controls.

### Statistics analysis

All experiments were performed with at least three independent biological replicates, and all data are presented as the mean ± standard deviation (SD). Statistical analysis of the data was conducted using Student’s t-test (for two-group comparisons) and one-way analysis of variance (ANOVA, for multiple-group comparisons). Statistical significance was indicated by asterisks: *P < 0.05 and **P < 0.01 or different letters.

## Results

### Sequence characterization and subcellular localization of SmMYB44

The nucleotide sequence of the *SmMYB44* gene was retrieved from the Eggplant Genome Database. Using the full-length cDNA of *‘*Lanshan Hexian*’*, a photosensitive eggplant cultivar, as the template, we obtained the complete gene sequence via PCR amplification and subsequent cloning validation. Sequencing results revealed that *SmMYB44* contains an intact 1110 bp open reading frame without stop codon insertion, which encodes a polypeptide consisting of 369 amino acid in plants, the amino acid sequences of R2R3-MYB proteins are conserved. Conserved domain analysis demonstrated that this protein contains a canonical R2R3-MYB domain and is thus classified into the R2R3-MYB transcription factor family. Additionally, SmMYB44 exhibited high homology and similarity with previously reported R2R3-MYB genes that negatively regulate anthocyanin biosynthesis in plants. Furthermore, we identified a C2/EAR motif (with the consensus sequence LxLxL or DLNxxP) in SmMYB44 (**Figure 1A**); as the EAR motif is a well-characterized core transcriptional repressor motif in plants, SmMYB44 is speculated to possess analogous transcriptional regulatory functions. Phylogenetic analysis demonstrated that SmMYB44 had the closest phylogenetic relationship with StMYB44 of potato (**Figure 1B**).

**Figure 1:**
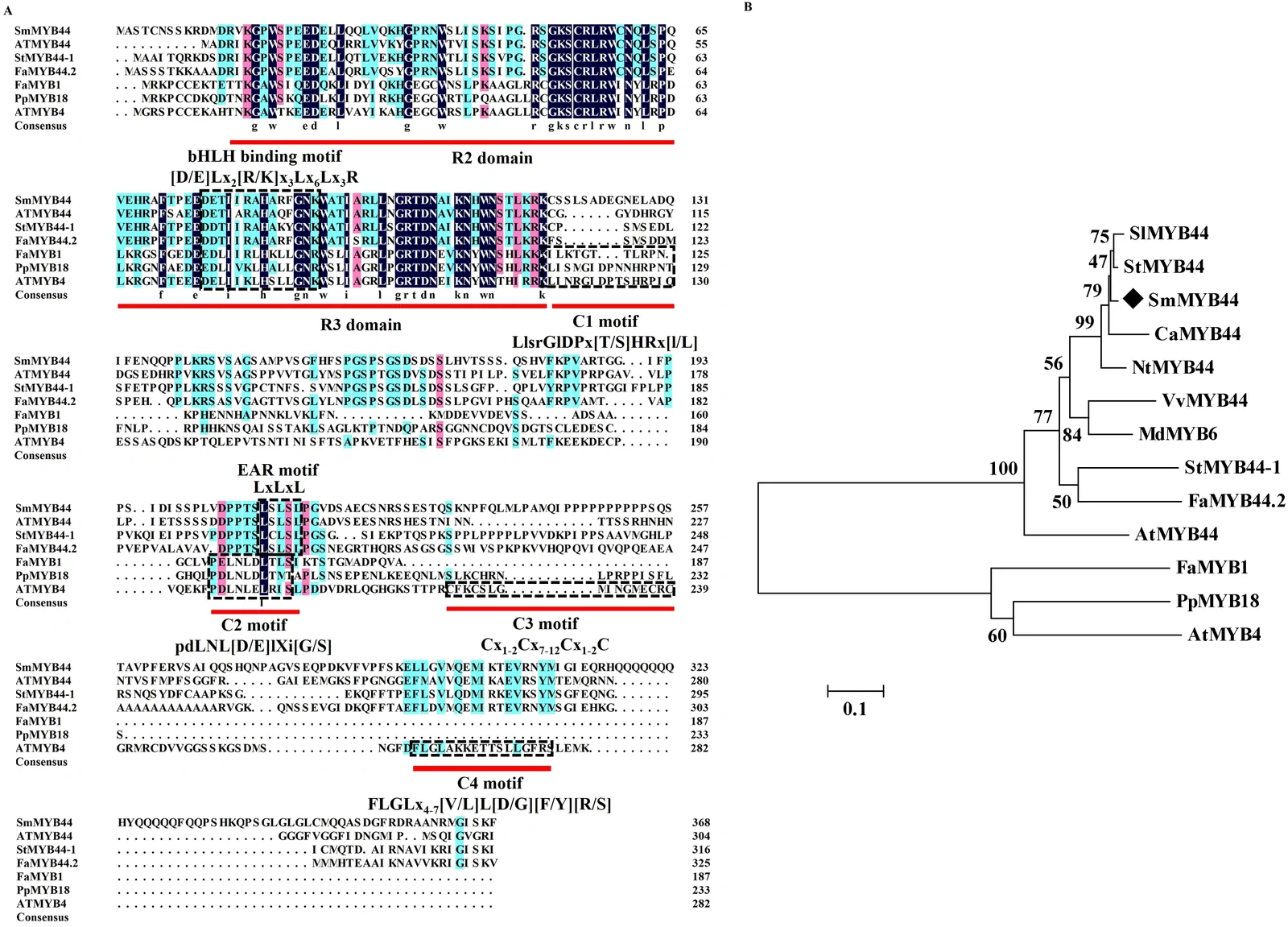
Amino acid sequence alignment analysis of SmMYB44. **(A)** Multiple sequence alignment of amino. acid sequence of SmMYB44 with other known R2R3-MYB TFs that negatively regulate anthocyanin biosynthesis in plants. SmMYB44 contained a conserved R2R3 MYB domain, and there was a C2/EAR motif (with the consensus sequence LxLxL or DLNxxP). **(B)** Phylogenetic analysis of the SmMYB44 protein with R2R3-MYB TFs from other species. The NCBI GenBank accession numbers of these sequences used in the figures are: SlMYB44 (XP_004236117.1), StMYB44 (MK410940), CaMYB44 (XM_016713322.2), NtMYB44 (XP_016487763.1), VvMYB44 (XP_002274094.1), MdMYB6 (AAZ20436.1), StMYB44-1 (MK410941), FaMYB44.2 (NP_001266963.1), AtMYB44 (NP_199791.1), FaMYB1 (AAK84064.1), PpMYB18 (XP_007211276.1), AtMYB4 (NP_195574.1).

The subcellular localization of SmMYB44 was predicted using WoLFPSORT (https://wolfpsort.hgc.jp), a specialized subcellular localization prediction tool, and the results indicated nuclear localization of this protein. Experimental validation in this study further confirmed the nuclear localization of SmMYB44, whereas the fluorescent protein in the control group was distributed in both the nucleus and cytoplasm. Combined with DAPI nuclear staining, the nuclear localization of SmMYB44 was unequivocally verified. Collectively, these results confirm that SmMYB44 is a nuclear protein (**Figure 2**).

**Figure 2:**
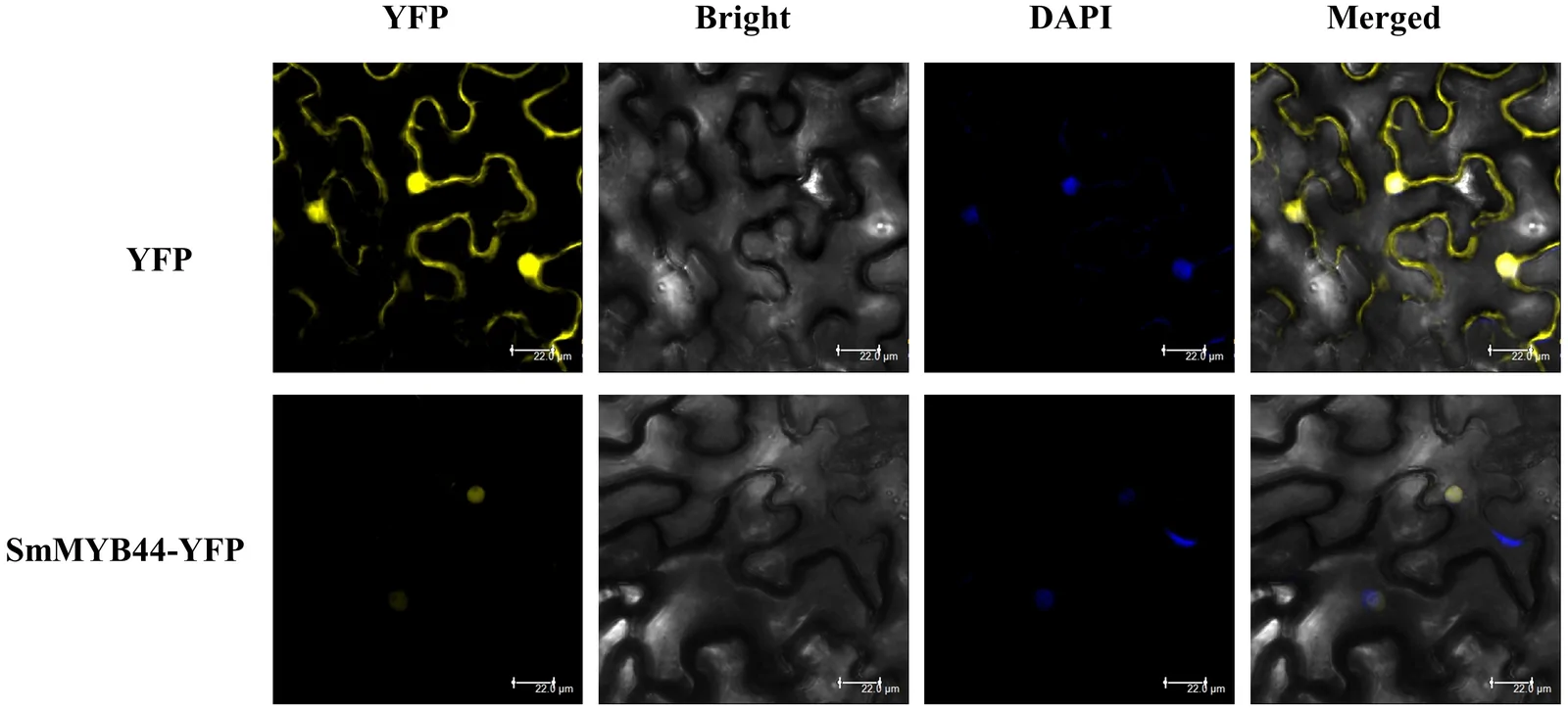
Subcellular localization of the SmMYB44 protein in *N. benthamiana*. The vector containing only YFP served as a control. The DAPI staining protocol was used to confirm the nucleus localization. Bars = 24.0 μm.

### Expression patterns of *SmMYB44* gene

The expression patterns of *SmMYB44* gene in eggplant was analyzed. While *SmMYB44* was expressed in various tissues of eggplant, with significant differences in expression levels. Compared to its highest expression in the peel, *SmMYB44* exhibits relatively high expression in roots and petals, whereas low expression levels were detected in flesh, septals, stems and leaves (**Figure 3A**). Our previously published study from Jiang et al. characterized anthocyanin contents across various tissues of eggplant cv. ‘Lanshan Hexian’. The highest anthocyanin concentration was observed in fruit peel, followed by petals, stems and leaves, while almost no anthocyanin accumulated in roots and flesh (**Figures 3A, B**). Combined with biochemical-assay data (Jiang et al., 2016), we found that the peel is the most active tissue for anthocyanin biosynthesis in eggplant, where the anthocyanin synthetic pathway is strongly activated. Meanwhile, *SmMYB44* is highly-induced in the peel to prevent excessive anthocyanin accumulation.

**Figure 3:**
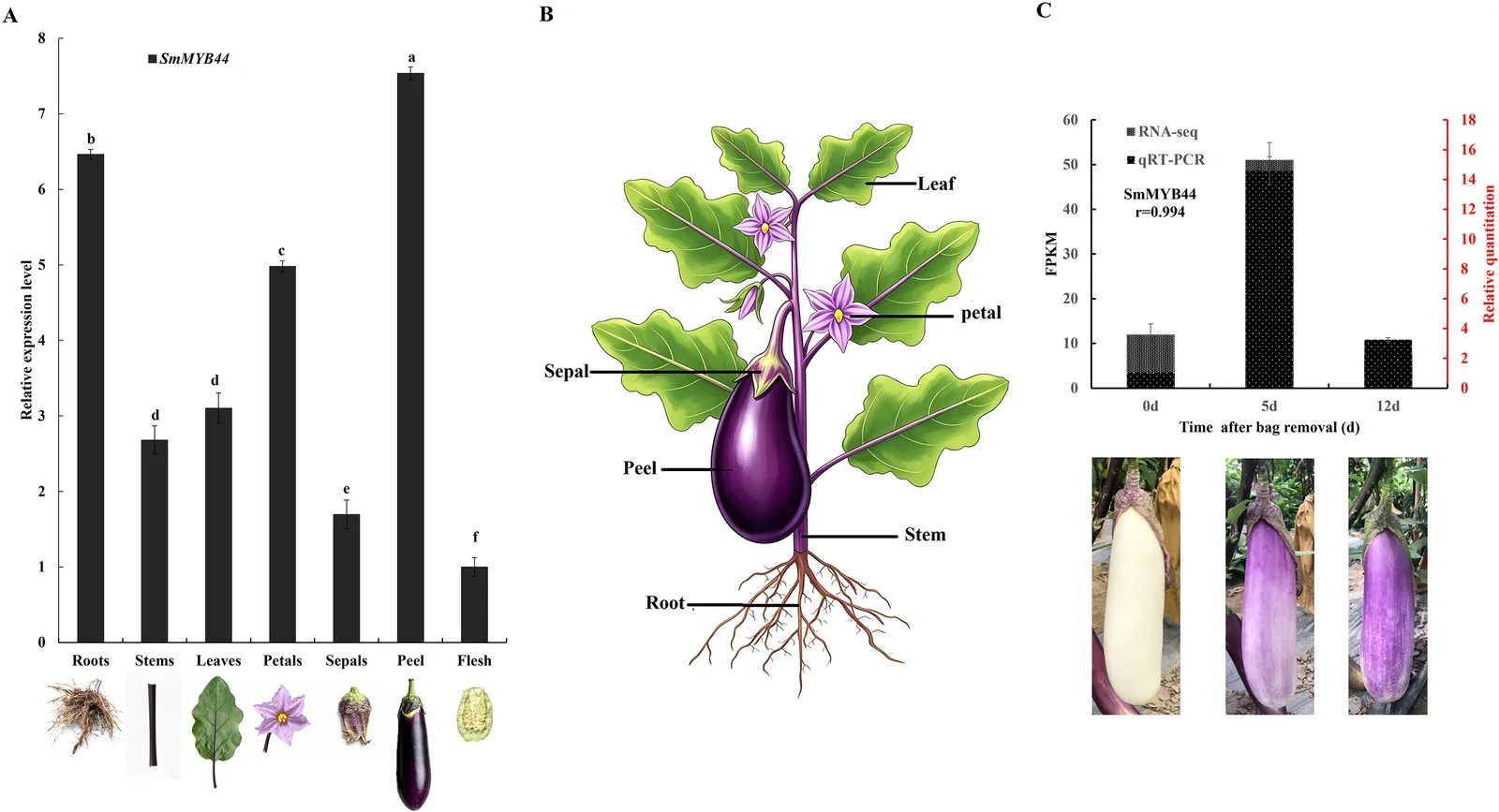
Tissue-specific expression patterns of *SmMYB44* in eggplant. **(A)** The tissue-specific expression of *SmMYB44* in eggplant roots, stems, leaves, petals, sepals, peel, and flesh, with different lowercase letters indicating significant differences at the p < 0.05 level. **(B)** Schematic diagram illustrating the different tissue parts of the eggplant plant. The eggplant cultivar ‘Lanshan Hexian’ exhibits dark purple fruit peel, purple leaf veins, stems, sepals and petals. **(C)** *SmMYB44* transcript levels at 0 d, 5 d, and 12 d after bag removal, determined by RNA-seq (FPKM) and RT-qPCR, show high concordance (r = 0.994). *SmMYB44* is weakly expressed in the dark, peaks at 5 d after light exposure, and declines by 12 d, indicating a light-responsive transient expression pattern. Lower panels show representative peel phenotypes at each time point.

Under shaded conditions, ‘Lanshan Hexian’ peel accumulated no anthocyanins and appeared milky white; after bag removal, purple pigmentation gradually deepened from 5 d to 12 d. Time-resolved transcriptomic profiling revealed that *SmMYB44* transcript abundance was minimal in darkness, increased sharply upon illumination to reach a maximum at 5 d, and declined by 12 d. RT-qPCR corroborated these findings, demonstrating strong concordance between RNA-seq and qRT-PCR datasets (Pearson correlation coefficient r = 0.994) (**Figure 2**). These results establish that *SmMYB44* is light-regulated and follows a transient induction kinetics during light-dependent anthocyanin accumulation in eggplant peel.

### SmMYB44 regulates anthocyanin biosynthesis in eggplant plants

In order to better understand the function of *SmMYB44*, *SmMYB44-OE* transgenic eggplant was obtained by *Agrobacterium tumefaciens*-mediated transformation of cotyledonary explants (**Supplementary Figure S3**). we generated six independent *SmMYB44-OE* lines. We extracted RNA from all positive plants and detected the relative expression level of *SmMYB44* via RT-qPCR. Three representative over-expression lines (*SmMYB44-OE*-1/-2/-3) were screened out according to the obvious expression-fold gradient of the target gene. Compared with WT, the fruit peels of *SmMYB44-OE* lines exhibited markedly attenuated purple pigmentation, accompanied by significantly reduced anthocyanin contents that were approximately one-third of those in the WT (P < 0.01) (**Figures 4A, B**). RT-qPCR detection verified strong *SmMYB44* over-expression within three transgenic lines, and the transcript abundance of *SmMYB44* was negatively correlated with anthocyanin accumulation (**Figure 4C**). Moreover, the transcription levels of all core anthocyanin-biosynthetic structural genes, including *SmCHI*, *SmCHS*, *SmF3H*, *SmDFR*, *SmF3’5’H* and *SmANS*, were significantly suppressed in *SmMYB44-OE* plants relative to WT (**Figure 4D**). Collectively, these phenotypic, physiological, and molecular results demonstrate that SmMYB44 functions as a negative regulator of anthocyanin biosynthesis in eggplant peel by repressing the transcription of structural genes.

**Figure 4:**
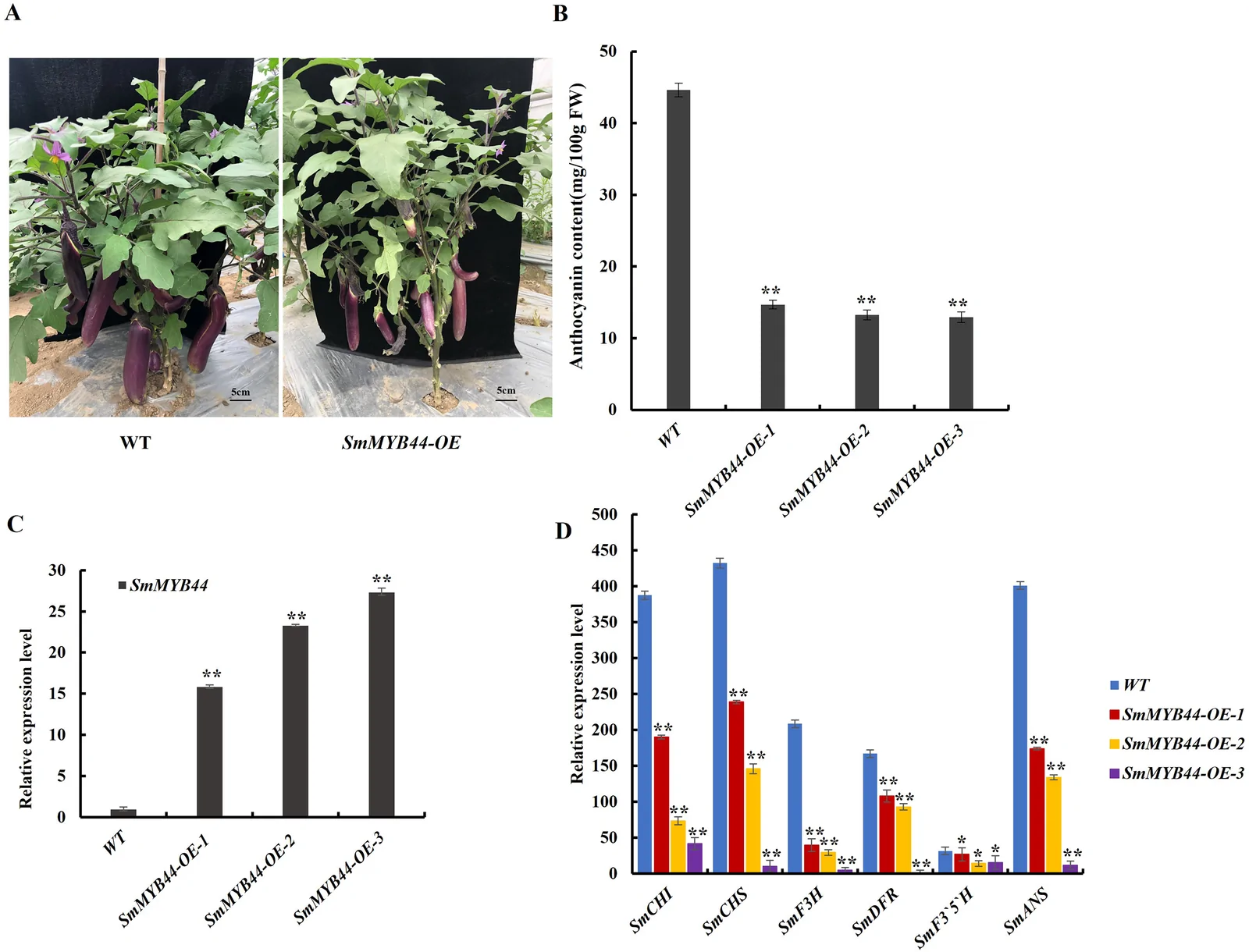
Effects of *SmMYB44-OE* transgenic eggplant on eggplant fruit coloration. **(A)** Phenotypic comparison of wild-type (WT) and *SmMYB44*-overexpressing (*SmMYB44-OE*) eggplant fruits. Scale bar, 1 cm. **(B)** Anthocyanin content in the peels of WT and three independent *SmMYB44-OE* transgenic eggplants. **(C)** RT-qPCR analysis the expression of *SmMYB44* in peel of WT and *SmMYB44-OE* transgenic eggplants. **(D)** RT-qPCR analysis the expression levels of anthocyanin biosynthetic structural genes *SmCHS* and *SmF3H* in WT and *SmMYB44-OE* transgenic eggplants. All data are presented as mean ± SD (n = 3). **indicates extremely significant difference compared with WT (**P < 0.01). SmMYB44 negatively regulates anthocyanin biosynthesis in eggplant. (*P < 0.05, **P < 0.01).

To further verify the biological function of *SmMYB44* in regulating anthocyanin biosynthesis, a VIGS vector *TRV2-SmMYB44* was constructed and transiently transformed into the peel of the eggplant cultivar *‘*116*’*. Phenotypic observation showed that *SmMYB44*-silenced eggplant peels displayed extensive anthocyanin accumulation and deeper purple coloration compared with the empty *TRV2* control (**Figure 5A**). Compared to *TRV2*, the anthocyanins contents in peel of *SmMYB44-*silenced eggplants were markedly increased (**Figure 5B**). RT-qPCR analysis confirmed that the transcript level of *SmMYB44* was prominently suppressed in silenced eggplant fruits, verifying the effective silencing of the target gene (**Figure 5C**). Furthermore, all key anthocyanin biosynthetic structural genes, including *SmCHI*, *SmCHS*, *SmDFR*, *SmF3’5’H*, *SmF3H* and *SmANS*, were significantly up-regulated in *SmMYB44*-silenced peels relative to the control, among which *SmF3H* showed the most dramatic induction (**Figure 5D**). These results further confirm that SmMYB44 acts as a negative regulator to anthocyanin biosynthesis in eggplant.

**Figure 5:**
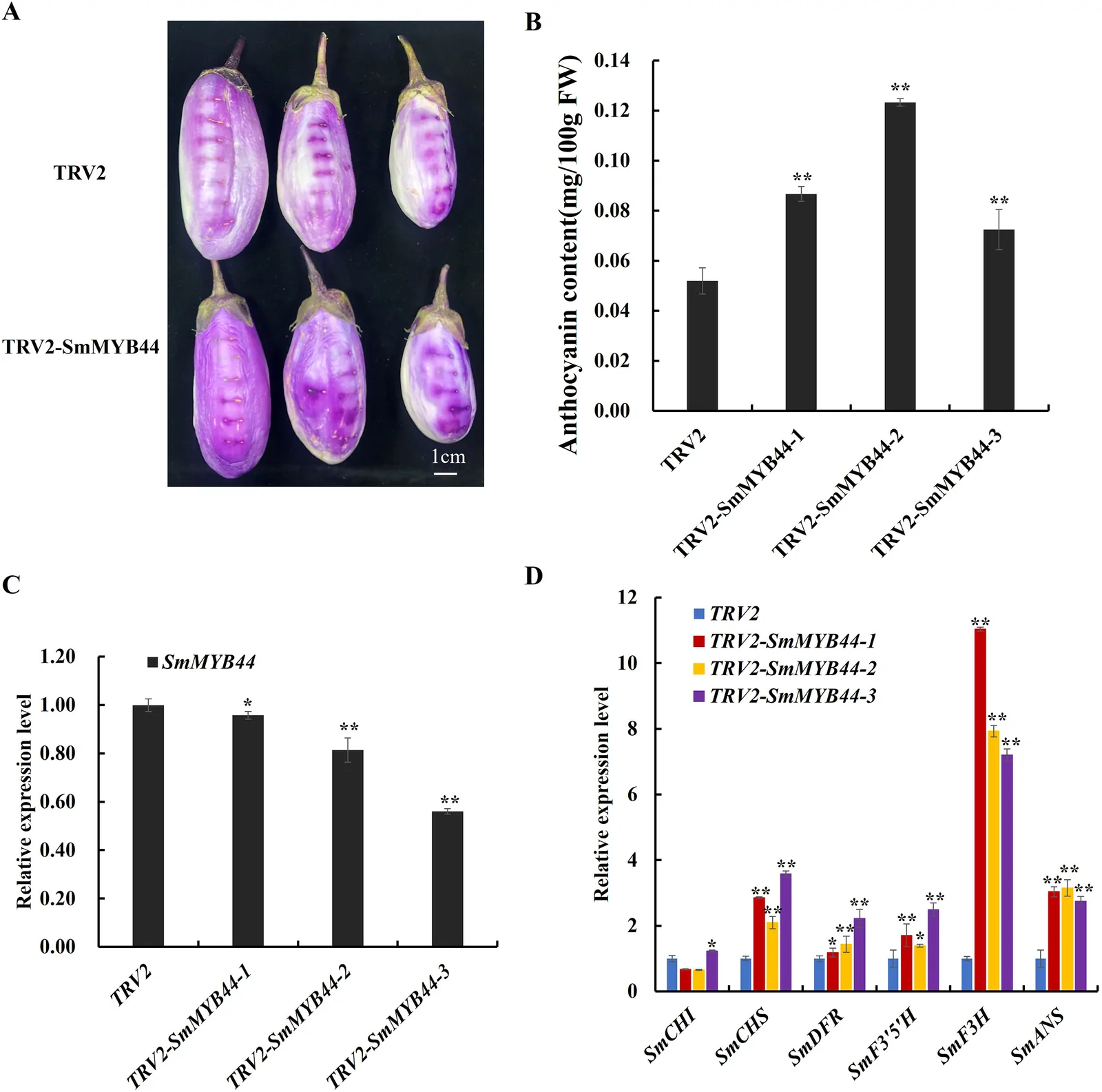
The effect of *SmMYB44* silencing in VIGS eggplants. **(A)** Phenotypic differences in *SmMYB44-*silenced eggplants. **(B)** Quantitative analysis of anthocyanins contents of peel from *SmMYB44*-silenced eggplants **(C)** The expression of *SmMYB44* in *SmMYB44*-silenced eggplants. **(D)** Expression of key anthocyanin synthesis genes *SmCHS* and *SmF3H* in *SmMYB44*-silenced eggplants. Values are means ± SD from three biological replicates. According to one-way analysis of variance was used to test the statistical significance. Significance was indicated by asterisks (*P < 0.05, **P < 0.01).

### Stable *NtMYB44*-edited tobacco lines with enhanced anthocyanin accumulation

The CRISPR/Cas9 editing vector targeting *NtMYB44* was introduced into tobacco K326 (*Nicotiana tabacum* cv. K326) explants via Agrobacterium-mediated genetic transformation (**Supplementary Figure S4**). Following double screening with hygromycin and cephalosporin, 13 hygromycin-resistant plants were obtained. Next-generation sequencing (NGS) was performed on the target site of the *SmMYB44* homologous *gene LOC107767835*(*NtMYB44)*, with 2 plants set as wild-type controls. The results indicated that 11 plants exhibited targeted mutations, yielding an editing efficiency of 84.62%. All mutant lines displayed biallelic mutations, comprising 2 biallelic homozygous mutant lines and 9 biallelic heterozygous mutant lines (**Supplementary Table S2**). Target site mutation types were diverse, with 1 bp short fragment insertions/deletions being the primary type. Additionally, complex mutations, including multi-base substitutions, large fragment deletions, and insertion-deletion-substitution complexes, were detected. Notably, all mutations occurred within the core region of the sgRNA target sites, consistent with the cleavage characteristics of the CRISPR/Cas9 system.

To investigate the effect of *SmMYB44* editing on anthocyanin accumulation in tobacco, phenotypic and physiological index detection revealed that the petals of T1 generation homozygous editing lines exhibited the typical pink color of the K326 variety 8–10 weeks after transplanting in a solar greenhouse. In contrast, the petal color of *SmMYB44* homozygous editing lines was significantly deepened, displaying a purple or deep red phenotype (**Figure 6A**). This phenotype was stably inherited in the homozygous lines. The results of the vanillin colorimetric method indicated that the anthocyanin content in the petals of T1 homozygous editing lines was significantly higher than that of the wild type (P < 0.01), with an increase of 2.5-4.2 times (**Figure 6B**). RT-qPCR results demonstrated that the expression of *SmMYB44* in the T1 homozygous edited lines was significantly down-regulated (P < 0.01) (**Figure 6C**). Following the inhibition of *SmMYB4*4 expression, the expression of key structural genes involved in anthocyanin synthesis, *NtCHS* and *NtF3H*, was significantly up-regulated (**Figure 6D**). This confirmed that SmMYB44 functions as a negative regulator of the anthocyanin synthesis pathway in tobacco, and its stable knockout can effectively promote anthocyanin accumulation in tobacco petals.

**Figure 6:**
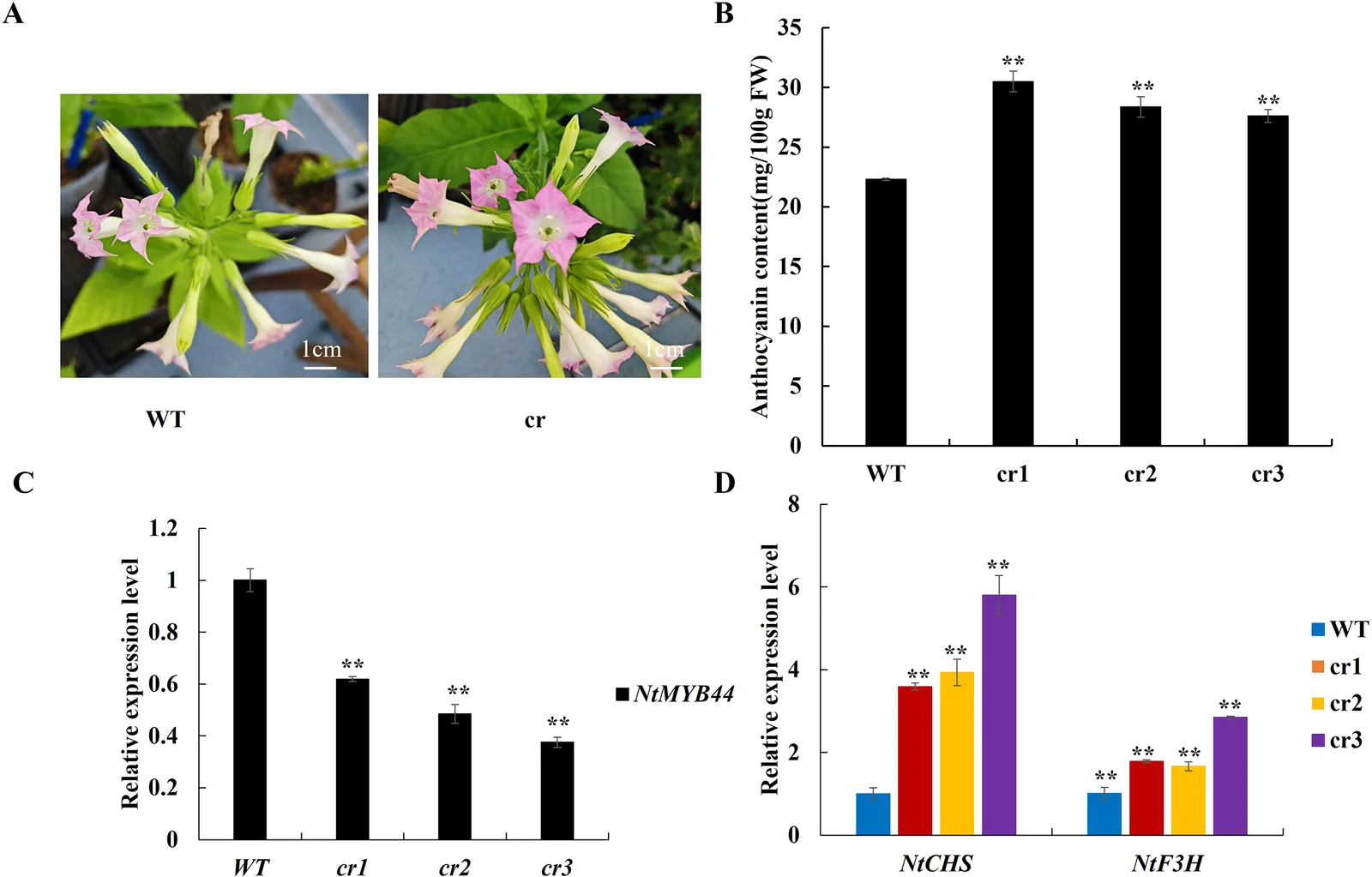
Effects of *SmMYB44* editing on anthocyanin accumulation in tobacco lines. **(A)** Phenotypic differences in *SmMYB44*-edited tobacco lines. **(B)** Quantitative analysis of anthocyanins contents of peel from *SmMYB44*-edited tobacco lines. **(C)** The expression of *SmMYB44* homologous gene *LOC107767835* in *NtMYB44*-edited tobacco lines. **(D)** Expression of key anthocyanin synthesis genes *NtCHS* and *NtF3H* in *SmMYB44*-edited tobacco lines. Values are means ± SD from three biological replicates. According to one-way analysis of variance was used to test the statistical significance. Significance was indicated by asterisks **(P < 0.01).

The T1 homozygous mutant lines were obtained through self-crossing. *NtActin7* was used as the internal reference gene, and the wild type served as the control. RT-qPCR results demonstrated that the expression of the *NtMYB44* was significantly inhibited (P < 0.01) in self-fertilized offspring lines, decreasing to 15%-20% of the wild-type level. The expression levels of key structural genes involved in anthocyanin synthesis were significantly up-regulated. Among these, late-synthesis genes *NtANS*, *NtCHI*, and *NtDFR* were up-regulated by 1.0-2.9 times; early-synthesis genes *NtF3’H* and *NtCHS* were up-regulated by 1.7-2.9 times and 3.9-10.8 times, respectively; and the key gene *NtF3’5’H* in the flavonoid synthesis branch pathway was up-regulated by 31.7-114.5 times (P < 0.01) (**Supplementary Figure S5**). These results indicate that the stable knockout of *SmMYB44* can relieve transcriptional inhibition of the anthocyanin synthesis pathway in tobacco.

### SmMYB44 suppresses the transcription of *SmCHS*, and *SmF3H* by binding to their promoters

To examine DNA trans-repression activity of SmMYB44, yeast one-hybrid assay was applied (**Figure 7B**). Only the combinations of LacZ-*CHSpro*/pB42AD-SmMYB44, and LacZ-*F3Hpro*/pB42AD-SmMYB44 showed blue color on the defective medium (-TU) with X-gal and succeeded in β-galactosidase expression. In contrast, other combinations and negative controls failed to express β-galactosidase. These results indicated that the SmMYB44 protein could directly bind to the promoters of *SmCHS* and *SmF3H*. Therefore, SmMYB44 may be involved in anthocyanin synthesis.

**Figure 7:**
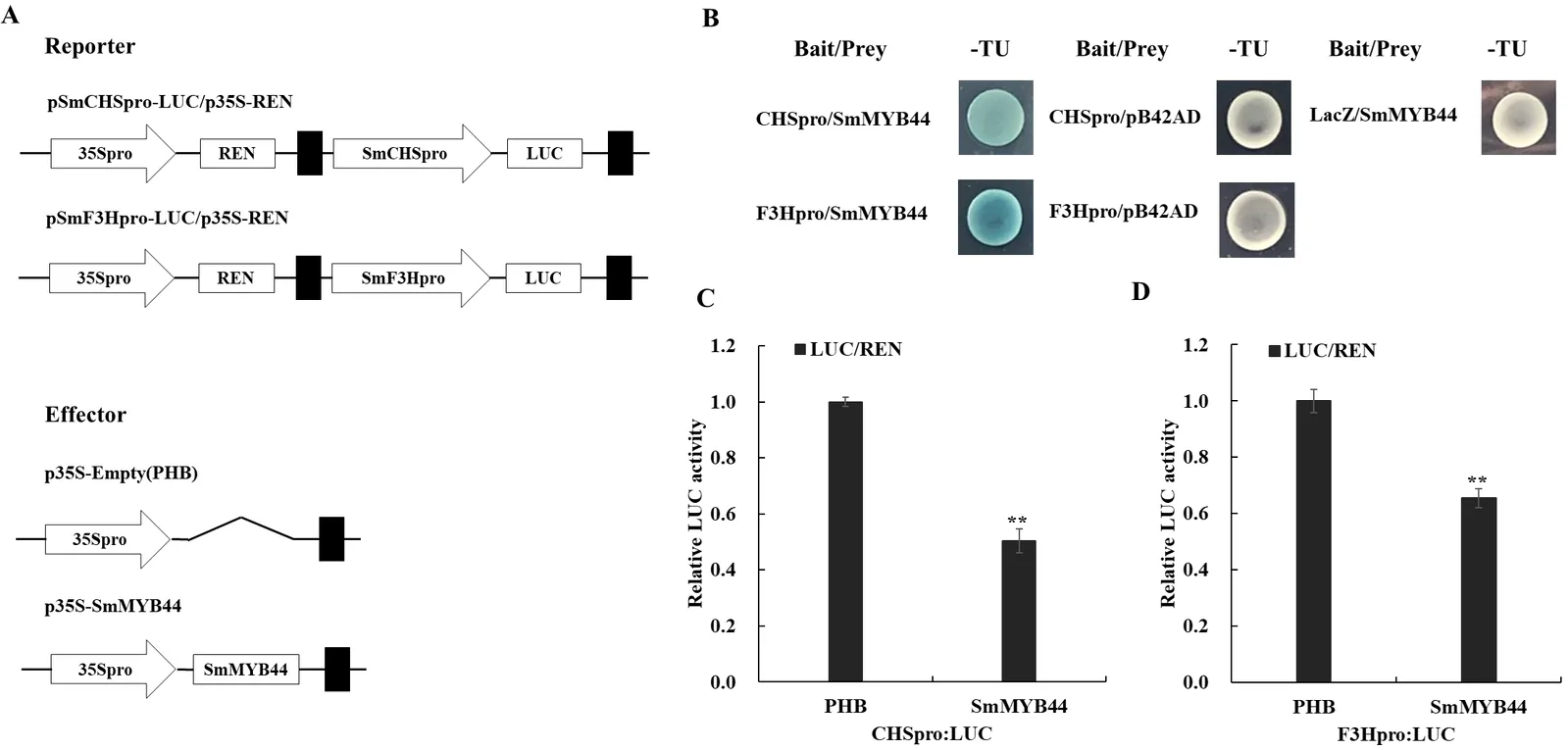
SmMYB44 represses the transcription of *SmCHS* and *SmF3H* via directly binding to their promoters. **(A)** Schematic diagram of the reporter and effector constructs used for dual-luciferase transient expression analysis. The reporter plasmid constructs contain the *SmCHS* or *SmF3H* promoter fused to LUC and REN luciferase driven by 35S promoter. The effector constructs contain the SmMYB44 driven by 35S promoter. **(B)** Specific binding of SmMYB44 to promoters of *SmCHS* and *SmF3H* by one-hybrid system. Positive transformants were cultured on SD/-Trp-Ura selective medium supplemented with X-gal at 30 °C for 3 d to observe yeast growth and color reaction. **(C)** Activation of *SmCHS* gene promoters by SmMYB44 in dual-luciferase assay. **(D)** Activation of *SmF3H* gene promoters by SmMYB44 in dual-luciferase assay. Data obtained via transient dual-luciferase assay in *Nicotiana benthamiana* leaves. The promoter activity of *SmCHS* and *SmF3H* promoters were quantified by calculating the LUC/REN fluorescence ratio. All data are presented as mean ± SD of three independent biological replicates. (**P < 0.01).

To determine the inhibitory activity of *SmCHS* and *SmF3H* gene promoters by SmMYB44, a dual-luciferase assay was conducted in *Nicotiana benthamiana* leaves. The reporter plasmid constructs contain the *SmCHS* or *SmF3H* promoter fused to LUC and REN luciferase driven by 35S promoter. The effector constructs contain the SmMYB44 driven by 35S promoter (**Figure 7A**). When the promoters of *SmCHS* and *SmF3H* were co-expressed with SmMYB44 protein, luciferase activity was significantly decreased (**Figures 7C, D**). These results reveal the binding specificity of SmMYB44 to the promoters of *SmCHS* and *SmF3H*, and its association with the suppression of promoter activities. we repeatedly conducted yeast one-hybrid screening against all core anthocyanin pathway genes including *SmCHI*, *SmDFR*, *SmANS* and *SmF3’5’H* using our existing experimental materials. The results confirmed that SmMYB44 only directly binds the promoters of early genes *SmCHS* and *SmF3H*, genetic materials from eggplant and tobacco exhibited coordinated expression changes across all anthocyanin structural genes.

### SmMYB44 interacts with the co-repressor SmTPL to jointly suppress the promoter activity of *SmCHS* and *SmF3H*.

To understand the roles of SmMYB44 in anthocyanin biosynthesis, Yeast two-hybrid assays showed interaction between SmMYB44 and SmTPL (**Figure 8A**). Recombinant plasmids pGBKT7-SmMYB44 and pGADT7-SmTPL were co-transformed into yeast competent cells. The combination of pGBKT7–53 and pGADT7-T served as the positive control, while empty pGBKT7 plus pGADT7 functioned as the negative control. All transformed yeast strains exhibited vigorous growth on SD/-Leu/-Trp medium, confirming successful plasmid transformation. Viable growth and blue staining were observed exclusively in the positive control and yeast cells co-transformed with pGBKT7-SmMYB44 and pGADT7-SmTPL on SD/-Leu/-Trp/-His medium supplemented with 1 mM 3-AT and SD/-Leu/-Trp/-His/-Ade medium containing X-α-Gal. No growth was detected in the negative control or cells transformed with pGBKT7-SmMYB44 and empty pGADT7, thereby confirming the specific interaction between SmMYB44 and SmTPL in yeast.

**Figure 8:**
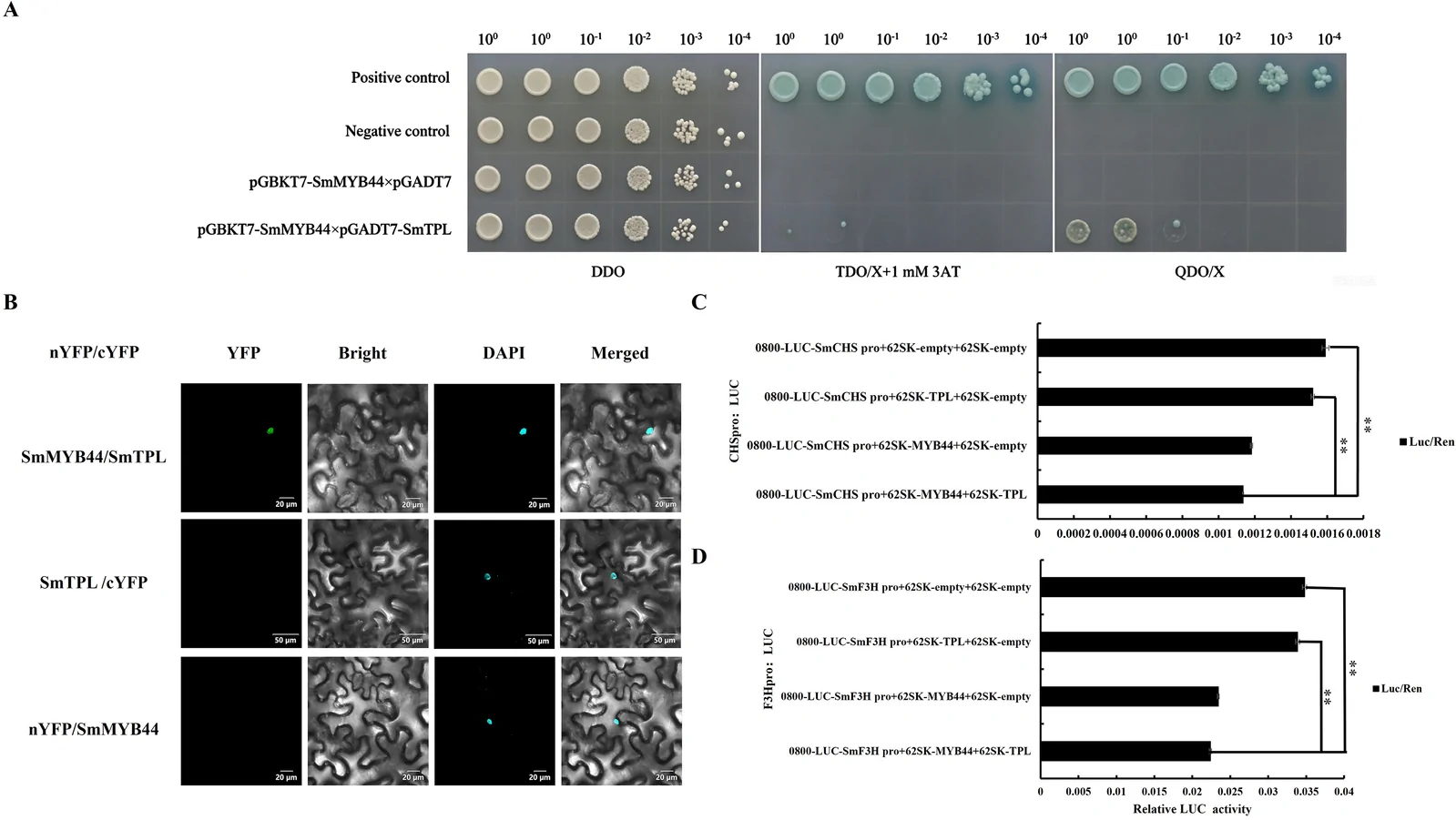
SmMYB44 interacts with SmTPL and inhibits its promotive role in *SmCHS* and *SmF3H* expression. **(A)** Analysis of the interaction between SmMYB44 and SmTPL by yeast two-hybrid assay. Growth on DDO, TDO/X + 1 mM 3AT, and QDO/X media confirmed protein interaction. Serial dilutions (10^0^-10^-4^) are shown, with positive/negative controls included. **(B)** BiFC assay showing SmMYB44 interact with SmTPL in *Nicotiana benthamiana* leaf epidermal cells. YFP, Yellow fluorescent protein field, Merged, overlay of BF and YFP field. Scale bars: 20 μm. **(C)** Activation of *SmCHS* promoter by SmMYB44 and SmTPL in Dual-LUC assay. **(D)** Activation of *SmF3H* promoter by SmMYB44 and SmTPL in Dual-LUC assay. The promoter activity of *SmCHS* and *SmF3H* promoter expressed as a ratio of LUC to REN. Values are means ± SD from six biological replicates. According to one-way analysis of variance was used to test the statistical significance. Significance was indicated by asterisks (*P < 0.05, **P < 0.01).

To further verify this interaction, BiFC assays were conducted in tobacco leaves (**Figure 8B**), Strong yellow fluorescent protein fluorescence, colocalized with DAPI nuclear staining, was detected in epidermal cells co-expressing SmMYB44-nYFP and SmTPL-cYFP. No fluorescent signal was observed in negative control groups (SmTPL-cYFP + nYFP, SmMYB44-nYFP + cYFP), demonstrating that SmMYB44 specifically interacts with SmTPL in the nucleus of tobacco cells.

To verify whether SmTPL affects the structural genes of anthocyanin synthesis in eggplant, a yeast one-hybrid experiment was conducted. The results indicated that SmTPL did not bind to the promoters of *SmCHS* and *SmF3H* (**Supplementary Figure S6**). Consistently, dual-luciferase data showed that sole expression of SmTPL exerted no observable influence on *SmCHS* and *SmF3H* promoter activity (**Figures 8C, D**). In contrast, SmMYB44 alone significantly repressed the transcriptional activity of both promoters. Co-transformation of SmMYB44 together with SmTPL yielded a consistent downward shift of LUC/REN ratios across all six biological replicates, revealing a synergistic repressive tendency of the SmMYB44-SmTPL complex, even though the difference between the SmMYB44 single group and co-expression group did not reach the threshold of P < 0.01 (**Figures 8C, D**). Specifically, the activity of the *SmCHS* promoter was significantly lower than that of the control group, and the activity of the *SmF3H* promoter was also significantly lower than that of the control group. Collectively, combined with yeast two-hybrid and BiFC evidence confirming their physical nuclear interaction, we conclude that SmMYB44 recruits SmTPL to assemble a repressive complex to strengthen transcriptional inhibition of downstream structural genes.

These findings indicate that SmMYB44 significantly suppresses the promoter activities of key structural genes in anthocyanin synthesis, and this repression is synergistically enhanced by SmTPL. SmMYB44 forms a transcriptional repressor complex with co-repressor SmTPL to negatively regulate the transcription of downstream structural genes in the anthocyanin biosynthesis pathway, thereby ultimately inhibiting anthocyanin biosynthesis.

## Discussion

Anthocyanin biosynthesis in horticultural crops is a sophisticated, light-dependent regulatory process controlled by conserved structural genes and transcription factor networks, which determines fruit color quality and environmental adaptability (Lasmar Dos Reis et al., 2026). As core regulatory components, R2R3-MYB transcription factors dominate the transcriptional reprogramming of anthocyanin metabolism, including both activator and repressor members that precisely balance pigment accumulation to avoid excessive metabolic consumption and photodamage (Zhou et al., 2019). Extensive studies have systematically characterized the functions of positive MYB regulators in eggplant, such as SmMYB113, SmMYB5, SmMYB35, and SmMYB6.2, which activate downstream structural genes and promote light-induced anthocyanin accumulation (Li et al., 2022a; Li et al., 2024). However, the negative regulatory mechanisms, especially light-responsive MYB repressors in photosensitive eggplant germplasms, remain poorly understood.

In the present study, we systematically cloned and functionally characterized the R2R3-MYB repressor gene *SmMYB44* from the photosensitive eggplant cultivar *‘*Lanshan Hexian*’*, clarified its subcellular localization, tissue-specific and light-responsive expression patterns, and revealed a novel regulatory mechanism by which SmMYB44 interacts with the co-repressor SmTPL to synergistically inhibit anthocyanin biosynthesis. These findings complement the negative regulatory module of light-dependent anthocyanin synthesis in eggplant and provide key target genes for crop color improvement.

Gene structural characteristics and subcellular localization are essential prerequisites for transcription factor function. In this study, sequence analysis showed that SmMYB44 encodes a typical R2R3-MYB protein with conserved N-terminal R2R3 DNA-binding domains and a C-terminal EAR repression motif (LxLxL) (**Figure 1A**), which is a canonical functional feature of plant R2R3-MYB repressors (Chow et al., 2023). EAR motif-containing MYB proteins generally execute transcriptional inhibitory functions by recruiting co-repressor complexes to suppress downstream target gene transcription (Huang et al., 2024). Moreover, multiple conserved short motifs distributed at the C-terminus represent the hallmark feature of S22-subgroup MYB44 proteins, which sets them apart from anthocyanin-activating MYB75 and MYB113 family members. Phylogenetic analysis further revealed that SmMYB44 has the closest genetic relationship with potato StMYB44 (**Figure 1B**). Accumulated literature has demonstrated that potato StMYB44 functions to suppress tuber anthocyanin accumulation upon high-temperature stress (Liu et al., 2019). By inference, SmMYB44 in eggplant probably takes part in environmental-stress-triggered negative modulation of anthocyanin biosynthesis within fruit peel. Subcellular localization prediction via WoLF PSORT and transient expression verification confirmed that SmMYB44 is a nuclear-localized protein, consistent with the typical subcellular distribution of transcription factors that exert transcriptional regulatory functions in the nucleus (**Figure 2**). Combined with the conserved EAR repression domain, these structural and localization characteristics sufficiently support that SmMYB44 functions as a nuclear transcriptional repressor involved in anthocyanin metabolic regulation.

Spatio-temporal expression patterns largely determine the biological function of regulatory genes in plant metabolic pathways. The mRNA transcript abundance of *SmMYB44* forms a typical negative-feedback homeostatic regulatory loop with the final accumulation of anthocyanins in the eggplant cultivar ‘Lanshan Hexian’(**Figure 3**). Published biochemical quantification data confirm that the eggplant peel is the core organ for anthocyanin biosynthesis, exhibiting significantly higher total anthocyanin content than petals, stems, leaves, and other tissues. Consistently, the transcript level of *SmMYB44* peaks in peel tissue(**Figure 3A**). The coordinated variation of these two indicators reveals an endogenous balance regulatory mechanism. After the complete anthocyanin biosynthesis pathway in the fruit peel is strongly activated, the repressive transcription factor *SmMYB44* is induced and significantly upregulated. As a transcriptional inhibitory molecular switch, SmMYB44 attenuates the transcriptional activity of downstream structural genes by binding to their promoters, thereby constraining continuous pigment synthesis and preventing excessive deposition of anthocyanins. Root tissues maintain high transcript abundance of *SmMYB44* but accumulate negligible anthocyanins. Petals display moderate purple phenotypes, accompanied by medium transcript levels of *SmMYB44*, which enables fine-tuning of pigment concentration. Flesh tissue exhibits extremely low transcript abundance of *SmMYB44* and negligible anthocyanin deposition. Furthermore, core anthocyanin biosynthetic genes exhibit extremely low basal transcriptional activity in eggplant roots and flesh, which barely generate anthocyanin precursors and inherently lack the capacity for massive pigment production.

Similar light-mediated antagonistic regulation of MYB repressors has been reported in other horticultural crops, such as potato StMYB44 and pepper CaMYB108a, which are also suppressed by light signals to promote pigment accumulation (He et al., 2026). Although these repressors are downregulated by light in those species, our results reveal a distinct light-triggered negative feedback loop mediated by *SmMYB44* in photosensitive eggplant peel. The light signaling pathway co-activates positive regulators and the negative regulator of the anthocyanin pathway. During the early stage of light exposure, positive transcription factors predominate and rapidly activate anthocyanin biosynthesis. Subsequent accumulation of *SmMYB44* then slows pigment synthesis to prevent redundant anthocyanin deposition and maintain cellular homeostasis. This mechanism enables a rapid light response in eggplant and mitigates the metabolic burden and potential cytotoxicity associated with excessive anthocyanin accumulation. Numerous light-responsive cis-elements, exemplified by the G-box, GT1-motif, Box 4, GATA-motif, TCT-motif, chs-CMA1a and the light-related MYB-binding site MRE, are present in the *SmMYB44* promoter (**Supplementary Table S1**), supporting its light-inducible property. Notably, four canonical G-box motifs in the *SmMYB44* promoter act as binding sites for HY5, the core regulator of plant light signaling. We speculate that HY5 directly targets these G-box elements and activates SmMYB44 transcription under light conditions, thereby linking light signal perception to the feedback modulation of anthocyanin metabolism and explaining the light-inducible expression pattern of *SmMYB44* in eggplant peel.

CRISPR/Cas9 gene editing has become a precise and efficient technical tool for functional verification of regulatory genes and quality improvement of horticultural crops, and knockout of MYB repressors can effectively release the inhibition of anthocyanin synthesis and enhance fruit commodity value (Chen et al., 2022). At present, CRISPR editing of anthocyanin regulatory genes in eggplant is mainly concentrated on positive regulatory factors such as SmMYB1 and SmERF118, while gene editing research on MYB negative regulators remain scarce (Li et al., 2025). In this study, we successfully obtained stably edited tobacco lines of *NtMYB44* with high editing efficiency (**Supplementary Figure S4**). Knockout of *NtMYB44* significantly up-regulated the expression of anthocyanin synthetic structural genes (**Supplementary Figure S5**), greatly increased petal anthocyanin content, and produced stable color-enhanced phenotypes (**Figure 6**). Heterologous overexpression of *SmMYB44* in tobacco resulted in slightly enhanced pigmentation of petal relative to wild-type (**Supplementary Figure S1**). Transcriptome heatmap analysis revealed that overexpressing *SmMYB44* markedly repressed the transcription of core structural genes involved in anthocyanin biosynthesis, including *F3H*, *CHI*, *DFR* and *ANS* (**Supplementary Figure S2**). These results verify the application potential of *SmMYB44* as an excellent editing target for crop color improvement. Consistent with previous studies in tomato and potato, editing of MYB repressors can effectively activate anthocyanin metabolic pathways and improve fruit pigmentation. Therefore, *SmMYB44* can be used as a key target gene for CRISPR-mediated molecular breeding of eggplant peel color, providing new technical support for the genetic improvement of photosensitive eggplant germplasms.

Direct binding to structural gene promoters is the core transcriptional inhibitory mechanism of R2R3-MYB repressors. Previous studies have confirmed that multiple MYB repressors in plants, including Arabidopsis AtMYB4 and eggplant SmMYB86, directly bind to the promoters of early anthocyanin synthetic genes and inhibit their transcription, thereby blocking anthocyanin synthesis (Li et al., 2021; Banerjee et al., 2024). In this study, yeast one-hybrid and dual-luciferase assays verified that SmMYB44 could directly bind to the promoters of *SmCHS* and *SmF3H* and significantly inhibit their transcriptional activity (**Figure 7**). *In vivo* genetic evidence from overexpression and VIGS silencing further validated this regulatory function: *SmMYB44*-overexpressing eggplant lines showed significantly reduced peel anthocyanin content and down-regulated expression of anthocyanin synthetic genes (**Figure 4**), whereas transient silencing of *SmMYB44* significantly promoted pigment accumulation and activated structural gene expression (**Figure 5**). These results fully demonstrate that SmMYB44 negatively regulates eggplant anthocyanin biosynthesis by targeting early key synthetic genes, consistent with the inhibitory mode of typical EAR-motif-containing MYB repressors in horticultural crops (Ohta et al., 2001).

Notably, although yeast one-hybrid assays confirmed that SmMYB44 cannot directly bind to the promoters of late anthocyanin biosynthetic genes including NtDFR and NtANS, obvious down-regulation of these late-stage structural genes was detected in *NtMYB44*-edited tobacco lines (**Supplementary Figure S5**). On one hand, SmMYB44 directly represses early-step genes *SmCHS* and *SmF3H*, perturbing flavonoid metabolic flux; shifts in intermediate metabolite levels may trigger metabolic-feedback regulation of downstream late biosynthetic genes. on the other hand, SmMYB44 recruits the co-repressor SmTPL via its EAR motif, which may interfere with the MBW activator complex. As the master regulator of late anthocyanin genes, this interference could indirectly repress these genes without physical contact with their promoters. At this stage, we are unable to quantify the relative contribution of these two regulatory pathways. Further biochemical and genetic assays will be required to clarify how SmMYB44 modulates the expression of late anthocyanin structural genes.

Protein-protein interaction constitutes a critical auxiliary regulatory layer for MYB transcription factors to amplify or enhance their regulatory effects. TPL/TPR proteins are well-characterized universal co-repressors in plants that lack autonomous DNA-binding ability and must be recruited by EAR-motif-containing transcription factors to execute transcriptional repression (Huang et al., 2024). In apple, MdMYB44 interacts with MdTPR1 via its C-terminal EAR motif and represses the expression of *MdCCD4* and *MdCYP97A3* through histone deacetylation to balance fruit carotenoid profiles. By contrast, VvMYB44 in grape and IbMYB44 in sweet potato attenuate anthocyanin production indirectly by disrupting the assembly of MBW activator complexes via TPL ortholog recruitment (Li et al., 2020; Leng et al., 2025). Consistent with this established model, our yeast one-hybrid and dual-luciferase results demonstrated that SmTPL could not directly associate with or activate the promoters of *SmCHS* and *SmF3H*, eliminating the misleading inference that SmTPL functions as an activator of anthocyanin biosynthetic genes (**Figure 8**). Our protein interaction assays verified the physical interaction between SmMYB44 and SmTPL in the nucleus. This indicates that SmMYB44 relies on recruiting the co-repressor SmTPL to form a stable transcriptional repressor complex, thereby synergistically inhibiting the transcription of downstream structural genes and fine-tuning anthocyanin accumulation. This interaction mechanism enriches the regulatory model of MYB-TPL module in anthocyanin synthesis, which has been rarely reported in eggplant. Most previous studies focused on the competitive inhibition of MBW complexes by MYB repressors, while our study identifies a dual-layer inhibitory branch in eggplant, where SmMYB44 directly binds structural gene promoters while recruiting SmTPL for synergistic repression. This represents a novel MYB44-TPL functional mode in Solanaceae and constitutes the core innovation of the present work. Unlike apple MdMYB44-MdTPR1 that modulates carotenoid metabolism, the SmMYB44-SmTPL cascade characterized here specifically responds to light signals to precisely tune peel anthocyanin biosynthesis, expanding the functional diversity of MYB(EAR)-TPL repressive modules in different secondary metabolic and light signal pathways.

In summary, this study clarifies the molecular mechanism of SmMYB44 negatively regulating light-induced anthocyanin biosynthesis in eggplant: SmMYB44 is a light-responsive nuclear R2R3-MYB repressor, which directly binds to the promoters of *SmCHS* and *SmF3H*, and synergistically enhances transcriptional inhibition by recruiting the co-repressor SmTPL, thereby suppressing anthocyanin accumulation in eggplant peel. This study fills the research gap of light-mediated MYB negative regulatory pathway in photosensitive eggplant, improves the transcriptional regulatory network of eggplant anthocyanin synthesis, and provides valuable gene resources and theoretical basis for eggplant fruit color improvement and stress-resistant molecular breeding.

In this study, we identified that the SmMYB44-SmTPL module negatively regulates anthocyanin biosynthesis in photosensitive eggplant through direct transcriptional repression of early structural genes. However, several limitations remain. First, although the eggplant transformation system used here is suitable for overexpression, we failed to obtain CRISPR/Cas9 knockout lines in the ‘Lanshan Hexian’ background because of poor callus regeneration after editing. Therefore, the loss-of-function evidence in this study mainly comes from virus-induced gene silencing in eggplant, and the CRISPR/Cas9 editing of the tobacco ortholog *NtMYB44* only provides auxiliary cross-species validation rather than direct proof of endogenous eggplant gene function. Second, yeast one-hybrid and dual-luciferase assays indicated that SmMYB44 binds to the promoters of *SmCHS* and *SmF3H*, but we did not perform electrophoretic mobility shift assays (EMSA) to independently confirm the physical binding and exact cis-element occupancy due to technical limitations. Third, all transgenic functional assays (overexpression, VIGS, and tobacco editing) were carried out under normal light conditions, and dark-shading treatments of transgenic eggplant were not conducted. Thus, whether the repressive function of SmMYB44 is strictly light-dependent has not been genetically verified. In addition, the hypothesis that HY5 acts as an upstream activator of SmMYB44 is only supported by bioinformatic prediction of G-box and other light-responsive elements in the SmMYB44 promoter, without direct biochemical or genetic evidence.

Despite these limitations, the current evidence from tissue-specific expression analysis, light-responsive expression patterns, eggplant overexpression, VIGS silencing, tobacco CRISPR editing, and protein-DNA and protein-protein interaction assays supports the conclusion that SmMYB44 functions as a nuclear R2R3-MYB repressor in anthocyanin biosynthesis. In future work, we will optimize the eggplant CRISPR/Cas9 system to generate stable *SmMYB44* knockout lines for definitive loss-of-function analysis; perform EMSA to map the SmMYB44-binding sites in target promoters; conduct dark-shading experiments using transgenic lines to test whether *SmMYB44*-mediated repression is light-dependent; and generate HY5 transgenic eggplant lines and use yeast one-hybrid and dual-luciferase assays to verify the proposed upstream regulatory relationship and further clarify the light-signaling pathway upstream of SmMYB44.

## Conclusions

This study reveals that light-responsive SmMYB44 represses eggplant anthocyanin synthesis by directly targeting *SmCHS* and *SmF3H* promoters and assembling a SmMYB44-SmTPL inhibitory complex. Our work establishes a new negative regulatory module for light-dependent pigmentation, validated by eggplant homologous overexpression and tobacco heterologous CRISPR editing, and supplies an effective gene target for eggplant color improvement (**Figure 9**).

**Figure 9:**
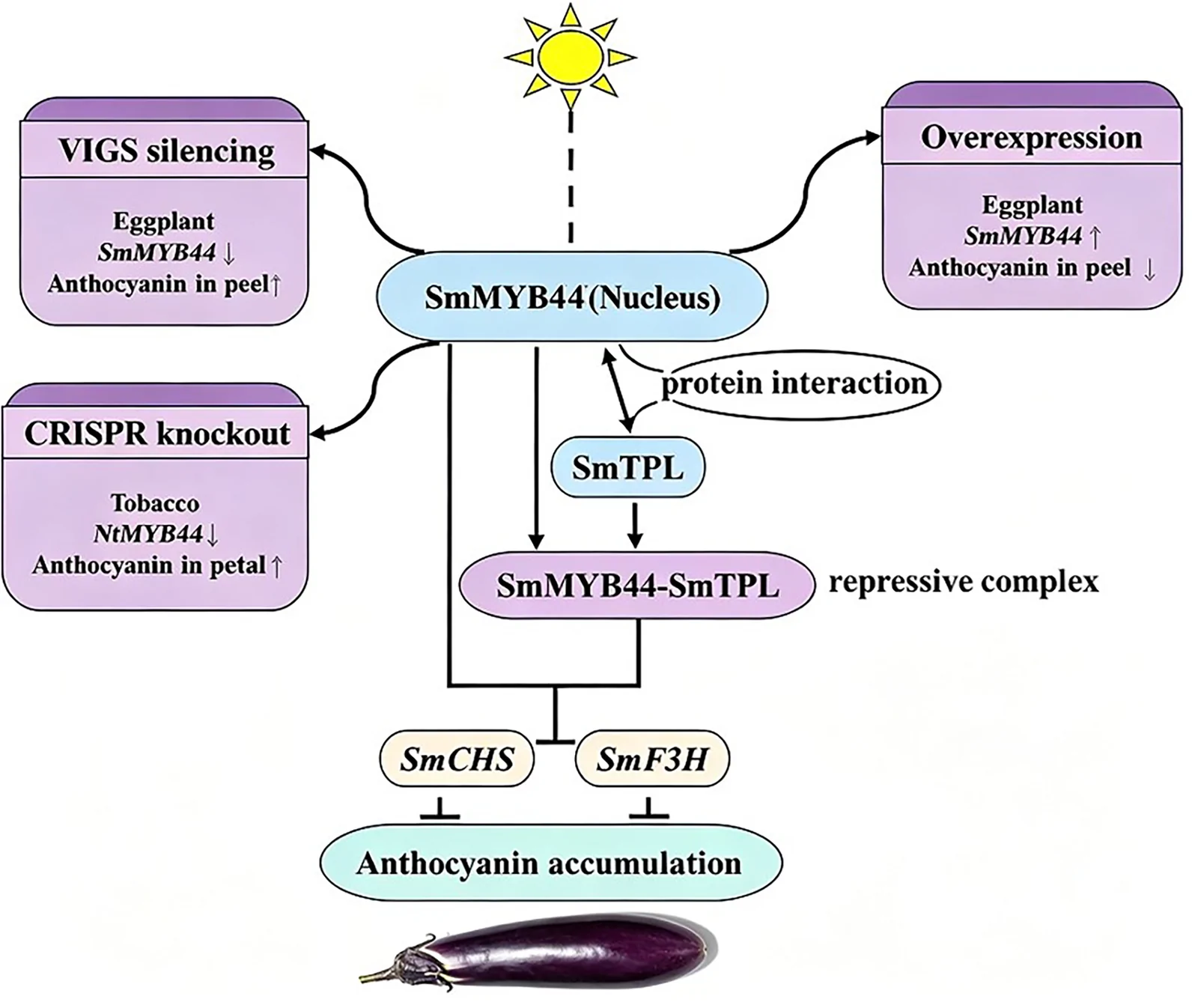
Working model of SmMYB44 negatively regulating anthocyanin biosynthesis in eggplant. Light signal induces the expression of *SmMYB44*. SmMYB44 interacts with the co-repressor SmTPL to form a transcriptional repression complex. This complex directly inhibits the transcription of anthocyanin early biosynthetic genes *SmCHS* and *SmF3H*, ultimately reducing anthocyanin accumulation in eggplant peel. Overexpression of *SmMYB44* decreases anthocyanin accumulation in eggplant peel; VIGS-mediated silencing of *SmMYB44* increases anthocyanin accumulation in eggplant peel; CRISPR-Cas9 knockout of tobacco ortholog *NtMYB44* elevates anthocyanin level in tobacco petals. Dashed lines indicate predicted regulatory relationships.

## Data Availability

The original contributions presented in the study are publicly available. This data can be found here: NCBI BioProject PRJNA1544301, Sequence Read Archive (SRA) SRP745783.
