# Supplementary material for: SmMYB44, a transcriptional repressor, negatively regulates anthocyanin biosynthesis in eggplant (Solanum melongena L.)
Source: Front Plant Sci. 2026 Oct 26;17:1927261. doi: 10.3389/fpls.2026.1927261 (PMC13750310; doi:10.3389/fpls.2026.1927261)
Supplement: Supplementary file 1 [file DataSheet1.docx]

**Fig S1. Acquisition *SmMYB44-OE* transgenic tobacco**

procedure to obtain *SmMYB44-OE* transgenic tobacco after Agrobacterium tumefaciens-mediated transformation of cotyledonary explants. Flower phenotype of wild-type and *SmMYB44-OE* tobacco. Obvious differences in petal pink pigmentation and floral morphology were observed between the two genotypes. Scale bars = 1 cm.

**Fig.S2 Expression patterns of the structural anthocyanin biosynthesis genes.**

Expression heatmap of anthocyanin biosynthesis structural genes in wild-type and *OE_SmMYB44* tobacco flower samples. The color scale represents the Log2 (fold-change to WT). Genes that are up-regulated appear in red, and those that are down-regulated appear in blue. Correspondence between gene locus IDs and gene abbreviations:*F3H*: (Flavanone 3-hydroxylase), LOC107806462: *F3H* (flavanone 3-hydroxylase), *DFR1/DFR2*: (Dihydroflavonol 4-reductase 1 / Dihydroflavonol 4-reductase 2), LOC107801774: *CHS* (chalcone synthase), LOC107825576: *CHI* (chalcone isomerase), LOC107810515: *CHI* (chalcone isomerase), LOC107826422: *CHS* (chalcone synthase), LOC107799879: *DFR* (dihydroflavonol 4-reductase), *ANS1/ANS2*: (Anthocyanidin synthase 1 / Anthocyanidin synthase 2), LOC107809744: *CHS* (chalcone synthase), LOC107803179: *CHS* (chalcone synthase), *CHI*: (Chalcone isomerase), LOC107788038: *MT*, LOC107786762: *PAL* (phenylalanine ammonia-lyase), LOC107785405: *4CL* (4-coumarate-CoA ligase), LOC107831213: *PAL* (phenylalanine ammonia-lyase).

**Fig S3. Acquisition *SmMYB44-OE* transgenic eggplant**

procedure to obtain *SmMYB44-OE* transgenic eggplant after Agrobacterium tumefaciens-mediated transformation of cotyledonary explants.

**Fig S4. Acquisition** ***NtMYB44*-edited tobacco lines**

procedure to obtain *NtMYB44*-edited tobacco lines after Agrobacterium tumefaciens-mediated transformation of cotyledonary explants.

**Fig. S5. Expression profiles of anthocyanin biosynthesis genes in** ***NtMYB44*-edited tobacco lines.**

Expression of key anthocyanin synthesis genes *NtCHI*, *NtCHS*, *NtDFR*, *NtF3`5`H*, *NtF3H* and *NtANS* in *NtMYB44*- edited tobacco lines. Values are means ± SD from three biological replicates. According to one-way analysis of variance was used to test the statistical significance. Significance was indicated by asterisks ** (P < 0.01).

**Fig S6. Specific binding of SmTPL to promoters of *SmCHS* and *SmF3H* by one-hybrid system.**

The results indicated that SmTPL did not bind to the promoters of *SmCHS* and *SmF3H*

**Table S1** **Cis-acting regulatory eleme****nts analysis of** ***SmMYB44* promoter sequences**

| **Motif name** | **Motif sequence** | **Position** | **+** | **Promoter Sequence Element Function Annotation** |
| --- | --- | --- | --- | --- |
| CGTCA-motif | CGTCA | 1671 | - | cis-acting regulatory element involved in the MeJA-responsiveness |
| P-box | CCTTTTG | 1504 | + | gibberellin-responsive element |
| Box 4 | ATTAAT | 608 | + | part of a conserved DNA module involved in light responsiveness |
| circadian | CAAAGATATC | 1168 | - | cis-acting regulatory element involved in circadian control |
| TGACG-motif | TGACG | 1671 | + | cis-acting regulatory element involved in the MeJA-responsiveness |
| TATC-box | TATCCCA | 186 | + | cis-acting element involved in gibberellin-responsiveness |
| chs-CMA1a | TTACTTAA | 633 | - | part of a light responsive element |
| GCN4_motif | TGAGTCA | 398 | - | cis-regulatory element involved in endosperm expression |
| GATA-motif | AAGATAAGATT | 1037 | - | part of a light responsive element |
| GATA-motif | GATAGGG | 1723 | - | part of a light responsive element |
| O2-site | GATGACATGG | 103 | - | cis-acting regulatory element involved in zein metabolism regulation |
| G-box | CACGTC | 112 | + | cis-acting regulatory element involved in light responsiveness |
| G-box | CCACGTAA | 837 | + | cis-acting regulatory element involved in light responsiveness |
| G-box | TACGTG | 838 | - | cis-acting regulatory element involved in light responsiveness |
| G-box | CACGAC | 973 | + | cis-acting regulatory element involved in light responsiveness |
| GT1-motif | GGTTAA | 6 | + | light responsive element |
| GT1-motif | GGTTAAT | 824 | - | light responsive element |
| GT1-motif | GGTTAA | 1343 | - | light responsive element |
| GT1-motif | GGTTAA | 1876 | - | light responsive element |
| MRE | AACCTAA | 1068 | - | MYB binding site involved in light responsiveness |
| MRE | AACCTAA | 1340 | - | MYB binding site involved in light responsiveness |
| ARE | AAACCA | 1403 | - | cis-acting regulatory element essential for the anaerobic induction |
| TCA-element | CCATCTTTTT | 988 | - | cis-acting element involved in salicylic acid responsiveness |
| TCA-element | CCATCTTTTT | 1699 | + | cis-acting element involved in salicylic acid responsiveness |
| TGA-element | AACGAC | 92 | - | auxin-responsive element |
| TCT-motif | TCTTAC | 1079 | - | part of a light responsive element |
| TCT-motif | TCTTAC | 1259 | + | part of a light responsive element |
| ABRE | ACGTG | 112 | - | cis-acting element involved in the abscisic acid responsiveness |
| ABRE | ACGTG | 838 | - | cis-acting element involved in the abscisic acid responsiveness |

**Table S2** **Target Site Mutations and Genotype Identification of *NtMYB44* in Gene-Edited Lines**

| **Line No.** | **Number of mutated chromosomes** | **Allele 1 mutant sequence (5‘-3’)** | **Allele 1 mutant sequence (5‘-3’)** | **Mutation type** | **Genotype classification** |
| --- | --- | --- | --- | --- | --- |
| 2 | 2 | TCCTTACCTTCATCAGCACCT--GA  GAGGAGGACTTCCT (1 bp deletion) | TCCTTACCTTCATCAaCACT--GAGAGGAGGACTTCCT (1 bp deletion + 1 bp substitution) | Deletion + Substitution | Biallelic heterozygous mutation |
| 3 | 2 | CCTTACCTTCATCAGCACCTAA (69 bp Deletion) GAAGCTCCGCTATAC (deletion) | CCTTACCTTCATCAGCACCTAAAAACATGCACCAAGAA (complex variation) | Large fragment deletion + Complex variation | Biallelic heterozygous mutation |
| 6 | 2 | CCTTACCTTCATCAGCACCT (a) AAGAGAGGAGGACTTCC (1 bp insertion) | CCTTACCTTCATCAGCACCTAA (t) GAGAGGAGGACTTCC (1 bp insertion) | Insertion | Biallelic heterozygous mutation |
| 11 | 2 | TTAACCTTCATCAGCAC--AaAAGAGGAGGACTTCCTCTT (2 bp deletion + 2 bp substitution) | TTAACCTTCATCAGCACaAA---AGGAAGACTTCCTCTTC (3 bp deletion + 3 bp substitution) | Deletion + Substitution | Biallelic heterozygous mutation |
| 33 | 2 | CCTTACCTTCATCAGCACCT (a) AAGAGAGGAGGACTTCC (1 bp insertion) | CCTTACCTTCATCAGCACCT (a) AAGAGAGGAGGACTTCC (1 bp insertion) | Insertion | Biallelic homozygous mutation |
| 34 | 2 | CCTTACCTTCATCAGCACCT (a) AAGAGAGGAGGACTTCC (1 bp insertion) | CCTTACCTTCATCAGCACCT (a) AAGAGAGGAGGACTTCC (1 bp insertion) | Insertion | Biallelic homozygous mutation |
| 37 | 2 | TTCATCAGCACCTAAaAaAaGaaGACTTCCTCTCAA (multiple base substitutions) | TTCATCAGCACCT (a) AAaAaAGGAGGACTTCCTCTCAA (1 bp insertion + multiple base substitutions) | Substitution + Insertion | Biallelic heterozygous mutation |
| 44 | 2 | TCCTTACCTTCATCAGCACCT--GAGAGGAGGACTTCCT (1 bp deletion) | TCCTTACCTTCATCAGCACCT (a) AAGAGAGGAGGACTT (1 bp insertion) | Deletion + Insertion | Biallelic heterozygous mutation |
| 46 | 2 | GCGAGTTCCCTTACCTTCATCAGCACCTAAaAGAGGAG (multiple base substitutions) | GCGAGTTCCCTTACCTTCATCgGCAt-------ACGgaGACTTC (multiple base deletion + substitution) | Substitution + Deletion | Biallelic heterozygous mutation |
| 47 | 2 | TCCTTACCTTCATCAGCACCT--GAGAGGAGGACTTCCT (1 bp deletion) | TCCTTACCTTCATCAGCACCT (a) AAGAGAGGAGGACTT (1 bp insertion) | Deletion + Insertion | Biallelic heterozygous mutation |
| 48 | 2 | CCTTCCCTTACCTTCATCAGCACCT (a) AAGAGAGGAGGAC (1 bp insertion) | CCTTCCCTTACCTTCATCAGCAC-------AGAGAGGAGGACTTCCT (4 bp deletion) | Insertion + Deletion | Biallelic heterozygous mutation |
| 43 | - | Control | Control | Wild-type | Wild-type |
| 45 | - | Control | Control | Wild-type | Wild-type |

Note:The wild-type sequence represents the original target site sequence of the *NtMYB44* gene.

WT Target Sequence (5'→3')：ACCTTCATCAGCACCTAAGAGAGG

The allele 1/2 mutant sequences are the target site sequencing results of CRISPR/Cas9-edited lines.

Lowercase letters indicate base substitutions and hyphens (-) indicate base deletions.

Control plant: refers to the unedited wild-type tobacco.

**Table S3 Sequences of primers used in this study**

| 引物名称  The name of the primer | 序列（5′→3′）  Sequence（ 5′–3′） |
| --- | --- |
| SmMYB44-F | GCCGCCTCCGTCTCAGTCTAC |
| SmMYB44-R | CTTGTCTGGCTGTTCGCTGACTC |
| PHB- *SmMYB44-*YFP-F | CTCTCTCTCAAGCTTGGATCCCGAGTACCTGTAACAGTTCC |
| PHB- *SmMYB44-*YFP-R | GCCCTTGCTCACCATACTAGTATCGAATTTGCTTATTCCCATG; CTCTCTCTCAAGCTTGGATCCATGGAAAATTCCAGTC |
| NtMYB44-F | GTGGAGAGGCTATTCGGCTATGACTG |
| NtMYB44-R | AGCTCTTCAGCAATATCACGGGTAGC |
| K5-KRSN-ccdb-F | CAGTGGTCTCATGCACCGGTAAATCTTGCCGGTTAGTTTCAG |
| K5-KRSN-ccdb-R | CAGTGGTCTCAAAACCTCTTAGTGCTGATGAAGGTTGCACC |
| pB42AD- *SmMYB44* -F | GATTATGCCTCTCCCGAATTCATGGCGAGTACCTGTAACAGTTCC |
| pB42AD- *SmMYB44*-R | AGAAGTCCAAAGCTTCTCGAGCTAATCGAATTTGCTTATTCCCATG |
| pB42AD- *SmTPL* -F | TGCCTCTCCCGAATTCATGGAATTTTGCAGATCT |
| pB42AD- *SmTPL* -R | TCCAAAGCTTCTCGAGGAAACCATTCAGTTTATT |
| Placzi- *SmCHS-pro*-F | TATTGGATCGGAATTCTCACTTTCATGATGCTGGCTTGC |
| Placzi- Sm*CHS*-pro-R | GAGCACATGCCTCGAGTCCATCCGCCACTAATGCTACCT |
| Placzi- Sm*F3H*-pro-F | TATTGGATCGGAATTCCTTTAATTATGTAGGTTTGGGTTGC |
| Placzi- Sm*F3H*-pro-R | GAGCACATGCCTCGAGTTATGATGGTCCGTGCTGTAT |
| pGreen-*CHSpro*-F | CTTGATATCGAATTCCTGCAGAGACACTCAACTGCATTC |
| pGreen-*CHSpro*-R | GCTCTAGAACTAGTGGATCCTGGTGAATAATATGCTACGGT |
| pGreen-*F3Hpro*-F | CTTGATATCGAATTCCTGCAGATTGAAGCTAAATTAAAC |
| pGreen-*F3Hpro*-R | GCTCTAGAACTAGTGGATCCCTTTTTCTTGTTTTTGGT |
| TRV2- *SmMYB44*-F | GTGAGTAAGGTTACCGAATTCTTATCTCCTCAGGTAGAGCATAGGG |
| TRV2- *SmMYB44*-R | CGTGAGCTCGGTACCGGATCCACTACCCGGACTGAAATGAAAACC |
| qRT – *SmMYB44*-F | GCCGCCTCCGTCTCAGTCTAC |
| qRT – *SmMYB44*-R | CTTGTCTGGCTGTTCGCTGACTC |
| qRT – SmCHI -F | CCTTGACGGGTAAGCAATACTCTG |
| qRT - SmCHI -R | GATGGAGGCACCATGTGGGAAGG |
| qRT – SmCHS -F | GGGAACAGTACTCCGGCTAGCC |
| qRT - SmCHS -R | AACACCTGAAATTGGGTCTGAACCA |
| qRT – SmF3H -F | GTGGTCCAAGACTGGCGTGAAAT |
| qRT – SmF3H -R | TTCTCTAACCCCATTGCTTCTGATA |
| qRT – SmF35H -F | TGGACCTCGTTGGAAGTTGCTAAG |
| qRT – SmF35H -R | TGCCATCGCGAACGTCAACATAT |
| qRT – SmANS -F | CTCGATTCCCACCTCGGACCTT |
| qRT – SmANS -R | TCAGCTGCAGCGTCCTGTTTGT |
| qRT – SmDFR -F | GGCCATTGAGACTTGCCGACAG |
| qRT – SmDFR -F | CACCATTGGTCAACTGTCCTGTACT |
| Actin-F | GTCGGAATGGGACAGAAGGATG |
| Actin-R | GTGCCTCAGTCAGGAGAACAGGGT |
| BD- *SmMYB44* -F  BD- *SmMYB44* -R  AD- *SmTPL* -F  AD- *SmTPL* -R  *SmMYB44*-cYFP-F  *SmMYB44*-cYFP-R  *SmTPL*-nYFP-F  *SmTPL*-nYFP-R | ATGGAGGCCGAATTCGCCGCCTCCGTCTCAGTCTAC  CAGGTCGACGGATCCCTTGTCTGGCTGTTCGCTGACTC  GAGGCCAGTGAATTCATGGAATTTTGCAGATCTGCTC  CAGCTCGAGCTCGATGGATCCTTATATGGAGCTCCGA  ATTACAGGTACCCGGGGATCCATGGCGAGTACCTGTAA  TGCCACCGCCGTCGACCTTGTCTGGCTGTTCGCTGACTC  GGACGCCGGCGGATCCATGGAATTTTGCAGATCTGCTCA AGCTCTGCAGGTCGACACCATAAAGAAACCATTCAGTTT |
| *NtActin*-F | ACCACTGCAGAACGGGAAAT |
| *NtActin*-R | CCGATCATGGATGGCTGGAA |
| qRT – *SmMYB44*-F | GTAACGAACTCGCCGATCAA |
| qRT – *SmMYB44*-R | TACCGGGACTGAAATGGAAAC |
| qRT - *NtANS*-F | GCCAAGCAGATCAGGAACCT |
| qRT - *NtANS*-R | GGTTGGGGGCATTTGGGATA |
| qRT - *NtCHI*-F | GTAGTATCGCCGAAAGAGTGTC |
| qRT - *NtCHI*-R | AATCACTGGATCGGAGGATTTC |
| qRT – *NtCHS*-F | GGTTTGGGAACTACTGGTGAA |
| qRT – *NtCHS*-R | AGTAGCAACACTGTGGAGAAC |
| qRT - *NtDFR*-F | CACTGGAGCAGCTGGATTTA |
| qRT - *NtDFR*-R | GTTCTCAGGATCACGAACAGTAG |
| qRT – *NtF3`5`H*-F | GCGAAAGGCTCAGTACAACTA |
| qRT - *Nt F3`5`H-*R | TTCTGCAAGTGCCCATTCT |
| qRT – *NtF3H*-F | AGCAGTGGTGAACTCGAATAG |
| qRT - *Nt F3H-*R | GCCTTCTCTCCTTCCCTAATTT |
| gene‑editing ‑F | ACCCAAATTAACAACCTCTGACT |
| gene‑editing *-*R | ATGAAGGCTCGAATCACTGT |

**The nucleotide sequence of** ***SmMYB44***

>Sme2.5_24183.1_g00002.1 /TE

atggcgagtacctgtaacagttccaagagagatatggatcgggttaaaggtccatggagc

cccgaagaggatgagcttttacagcagcttgttcagaaacatggccctagaaattggtcc

cttataagcaaatctatcccggggagatccggtaaatcttgccggttgcggtggtgtaat

cagttatctcctcaggtagagcatagggcttttactcccgaagaagatgagactattatt

cgggctcatgcccgattcgggaacaaatgggccaccatagcccgacttcttaatggccgg

actgataacgctattaagaaccactggaattctaccttgaagaggaagtgctcttcgctt

agtgccgatgaaggtaacgaactcgccgatcaaatttttgagaatcagcaaccgccgttg

aagagatccgtcagtgccggatccgctatgccggtttcgggttttcatttcagtccgggt

agtccgtcgggttcggatagtgattcgagtcttcatgttacgtcatcgtctcaatctcat

gtgtttaagcctgttgctagaaccggcggcatctttccgccgtcgattgatatttcatct

ccgcttgttgatcctcccacctctctcagcctttcgcttcccggtgttgactcagccgag

tgctcgaaccgttcgtccgaatcgactcagtcgaaaaatcctttccagttgatgcttccg

gcgatgcagattcctccaccaccaccaccgccgccgcctccgtctcagtctacggcggtg

ccgttcgaacgagtttctgcaatccaacagtctcatcagaaccctgccggagtcagcgaa

cagccagacaaggttttcgtgccgtttagcaaggagttgttaggtgtgatgcaggagatg

ataaagacggaggtgaggaattacatgattggaattgaacaaaggcatcagcaacagcag

cagcagcagcattatcaacaacagcagcagtttcagcaaccaagtcataaacagccaagt

ggtttagggcttggcttgtgtatgcaacaagcttctgatggattcagggatagggctgct

aatcgcatgggaataagcaaattcgattag

**The translated sequence of SmMYB****44**

>Sme2.5_24183.1_g00002.1 /TE

MASTCNSSKRDMDRVKGPWSPEEDELLQQLVQKHGPRNWSLISKSIPGRSGKSCRLRWCN

QLSPQVEHRAFTPEEDETIIRAHARFGNKWATIARLLNGRTDNAIKNHWNSTLKRKCSSL

SADEGNELADQIFENQQPPLKRSVSAGSAMPVSGFHFSPGSPSGSDSDSSLHVTSSSQSH

VFKPVARTGGIFPPSIDISSPLVDPPTSLSLSLPGVDSAECSNRSSESTQSKNPFQLMLP

AMQIPPPPPPPPPPSQSTAVPFERVSAIQQSHQNPAGVSEQPDKVFVPFSKELLGVMQEM

IKTEVRNYMIGIEQRHQQQQQQQHYQQQQQFQQPSHKQPSGLGLGLCMQQASDGFRDRAA

NRMGISKFD

**The nucleotide sequence of *SmTPL***

>Sme2.5_01513.1_g00008.1
atggaattttgcagatctgctcatacacgggtttttcatacttgttctgagcttgaagac
gagttagtagcaatgacatctctcagtagagagcttgtattcttgattttacaatttcta
gacgaggaaaagttcaaagaaacagttcacaagcttgagcaagaatctggattcttcttt
aacatgaagtactttgaagatgaagtgcataatggtaactgggatgaagttgaaaagtat
ctttctggttttaccaaagtggatgataaccgttattccatgaaaatattctttgaaata
aggaagcaaaagtatcttgaggcattggacaagcgtgaccggtccaagggtgttgaaatt
cttgtaaaggatcttaaagtttttgcatctttcaatgaagagcttttcaaggagataact
cagctgttgacactagagaatttcagggagaatgaacagctgtccaagtatggagatact
aaatctgcacgagctattatgttggttgagctcaaaaagcttattgaagcaaatcccctg
tttcgtgacaaattgcaatttcccaacctcaaaaattcaaggttacgaacgttgattaac
caaagcttaaattggcagcaccaactttgtaaaaatcctaggccaaatccagatatcaaa
acactttttgtggatcattcctgtggacaaccaaatggtgcccgagctccatcacctgca
aataatccgttgcttggatcagtgcctaaaccaggcagcttccctcctcttggtgcacat
gggcctttccaacctgggccagcacctgtggcagctcctcttgctggctggatgtccaac
ccacccactgtagctcatccagctgtttctggtggacctatgggtcttggtccttcatct
attccaggaggtcacgtgttcaagctttggaaacagcctctggcagaaatgcaaggtaag
gctgcgtacaatagacccttgtggtggagcccttcttcggatcctgtgccttctcttaaa
catcctaggactccaccaacaaatccctctattgattatccatctggggaatcggatcat
gctaccaaaagaactaggtcactgggaatatctgatgaggtcaatcttcctgtgaatgtg
ctgcctatatcatttccagggcaaggccataatccatctcttagtgtacctgatgacttg
cccaagactgttgcaagaatgttaaaccagggatcctctcccatgagcatggatttccat
ccttctcagcagacattgcttctagttggcacgaatgttggagatattgcattgtgggaa
gttggttcaagggagagactggtgttgagaaacttcaaagtctgggacttgggtgcatgt
tcaatgccattacagactgctctggtaaaagatcctggtgtctcagttaaccgtgttata
tggagctccgatggttcgttatttggggttgcatactctaggcacattgttcaaatatat
tcctatcacgggaatgatgacatacgtcagcatgtggagattgatgctcatgttgggggt
gtaaatgatcttgcattctctcaccctaataagcagctctgtgttataacatgtggagat
gacaaaactataaaggtttgggatgccacgtctggtgcaagacaatatacatttgagggt
catgaagcacctgtatactctgtgtgccctcaccataaagaaaccattcagtttattttc
tctacagcgttagatggaaagataaaagcgtggttatatgataatttgggatctcgagta
gattatgaggctcctggtcgttggtgcacaacaatggcatacagtgctgatggaactagg
ctgttttcgtgtggaaccagcaaagatggggagtcacacattgttgagtggaatgaaagt
gaaggggctgttaagagaacatatcaagggtttcggaaaagatcgctgggtgttgtacaa
tttgatacaactaaaaatcgttttctggcagctggtgatgatttctcaatcatggaagct
taccaaataacgaaccatcggagctccatataa

**The translated sequence of the R2R3-MYB family proteins from other plants**

>SmMYB44

MASTCNSSKRDMDRVKGPWSPEEDELLQQLVQKHGPRNWSLISKSIPGRSGKSCRLRWCNQLSPQVEHRAFTPEEDETIIRAHARFGNKWATIARLLNGRTDNAIKNHWNSTLKRKCSSLSADEGNELADQIFENQQPPLKRSVSAGSAMPVSGFHFSPGSPSGSDSDSSLHVTSSSQSHVFKPVARTGGIFPPSIDISSPLVDPPTSLSLSLPGVDSAECSNRSSESTQSKNPFQLMLPAMQIPPPPPPPPPPSQSTAVPFERVSAIQQSHQNPAGVSEQPDKVFVPFSKELLGVMQEMIKTEVRNYMIGIEQRHQQQQQQQHYQQQQQFQQPSHKQPSGLGLGLCMQQASDGFRDRAANRMGISKFD

>AtMYB44

MADRIKGPWSPEEDEQLRRLVVKYGPRNWTVISKSIPGRSGKSCRLRWCNQLSPQVEHRPFSAEEDETIARAHAQFGNKWATIARLLNGRTDNAVKNHWNSTLKRKCGGYDHRGYDGSEDHRPVKRSVSAGSPPVVTGLYMSPGSPTGSDVSDSSTIPILPSVELFKPVPRPGAVVLPLPIETSSSSDDPPTSLSLSLPGADVSEESNRSHESTNINNTTSSRHNHNNTVSFMPFSGGFRGAIEEMGKSFPGNGGEFMAVVQEMIKAEVRSYMTEMQRNNGGGFVGGFIDNGMIPMSQIGVGRIE

>StMYB44-1

MAAITQRKDSDRIKGPWSPEEDELLQTLVEKHGPRNWTLISKSVPGRSGKSCRLRWCNQLSPQVEHRAFTPEEDDTIIRAHAKYGNKWATIARLLSGRTDNAIKNHWNSTLKRKCPSMSEDLSFETPQPPLKRSSSVGPCTNFSSVMNPGSPSGSDLSDSSLSGFPQPLVYRPVPRTGGIFPLPPPVKQIEIPPSVPDPPTSLCLSLPGSGSIEKPTQSPKSPPLPPPPLPVVDKPIPPSAAVMGHLPRSNQSYDFCAAPKSGEKQFFTPEFLSVLQDMIRKEVKSYMSGFEQNGICMQTDAIRNAVIKRIGISKIE

>FaMYB44.2

MASSSTKKAAADRIKGPWSPEEDEALQRLVQSYGPRNWSLISKSIPGRSGKSCRLRWCNQLSPEVEHRPFTPEEDDTIIRAHARFGNKWATISRLLNGRTDNAIKNHWNSTLKRKFSSMSDDMSPEHQPLKRSASVGAGTTVSGLYLNPGSPSGSDLSDSSLPGVIPHSQAAFRPVAMTVAPPVEPVALAVAVDPPTSLSLSLPGSNEGRTHQRSASGSGSSWIVSPKPKVVHQPQVIQVQPQEAEAAAAAAAAAAAAARVGKQNSSEVGIDKQFFTAEFLDVMQEMIRTEVRNYMSGIEHKGMMMHTEAAIKNAVVKRIGISKVE

>FaMYB1

MRKPCCEKTETTKGAWSIQEDQKLIDYIQKHGEGCWNSLPKAAGLRRCGKSCRLRWINYLRPDLKRGSFGEDEEDLIIRLHKLLGNRWSLIAGRLPGRTDNEVKNYWNSHLKKKILKTGTTLRPNKPHENNHAPNNKLVKLFNKMDDEVVDEVSSADSAAGCLVPELNLDLTLSIKTSTGMADPQVA

>PpMYB18

MRKPCCDKQDTNRGAWSKQEDLKLIDYIRKHGEGCWRTLPQAAGLLRCGKSCRLRWINYLRPDLKRGNFAEDEEDLIVKLHALLGNRWSLIAGRLPGRTDNEVKNYWNSHLRRKLISMGIDPNNHRPNTFNLPRPHHKNSQAISSTAKLSAGLKTPTNDQPARSGGNNCDQVSDGTSCLEDESCGHQLPDLNLDLTMTAPLSNSEPENLKEEQNLMSLKCHRNLPRPPISFLS

>AtMYB4

MGRSPCCEKAHTNKGAWTKEEDERLVAYIKAHGEGCWRSLPKAAGLLRCGKSCRLRWINYLRPDLKRGNFTEEEDELIIKLHSLLGNKWSLIAGRLPGRTDNEIKNYWNTHIRRKLINRGIDPTSHRPIQESSASQDSKPTQLEPVTSNTINISFTSAPKVETFHESISFPGKSEKISMLTFKEEKDECPVQEKFPDLNLELRISLPDDVDRLQGHGKSTTPRCFKCSLGMINGMECRCGRMRCDVVGGSSKGSDMSNGFDFLGLAKKETTSLLGFRSLEMK

>SlMYB44

MAITTNSSKRDMDRVKGPWSPEEDELLQQLVQKHGPRNWSLISKSIPGRSGKSCRLRWCNQLSPQVEHRAFTPEEDETIIRAHARFGNKWATIARLLNGRTDNAIKNHWNSTLKRKCSSLSADEGNELADQLFENQQPPLKRSVSAGSAMPVTGFHFSPGSPSGSDSDSSLHVTSSSQSQVFKPVARTGGVFPPSIDISSPPVDPPTSLSLSLPGVDLAESSNRSADSTQSKNPFQWLLPPMQIPPPPPPPPPPLATTVPFERVSAIQQSLQNPDFGQNSGGEQPDKVFVPFSQELLGVMQEMIKTEVRNYMMGVEQKQQYQRQHYQQQQPQQFQQQNHQLPSGLGLGLCMQQASDCFRDRAANRMGLSKFD

>StMYB44

MDRVKGPWSPEEDELLQQLVQKHGPRNWSLISKSIPGRSGKSCRLRWCNQLSPQVEHRAFTPEEDETIIRAHARFGNKWATIARLLNGRTDNAIKNHWNSTLKRKCSSLSADEGNELADQIFENQQPPLKRSVSAGSAMPVSGFHFSPGSPSGSDSDSSLHVTSSSQSHVFKPVARTGGVFPQSIDISSPPVDPPTSLSLSLPGVDLAEFSNRSADSTQSKNPFQLLLPPMQIPPPPPPPPPPPPQATTVPFERVSAIQQSLQNPDFGKNAGGEQPDKVFVPFSQELLGVMQEMIKTEVRNYMMGVEQKQQFQQQQQHYQQQPQQFQQQNHQLPSGLGLGLCMQQASDGFRDRAANRMRLSKFD

>NtMYB44

MANSSNSSKKDMDRVKGPWSPEEDELLQQLVHKHGPRNWSLISKSIPGRSGKSCRLRWCNQLSPQVEHRAFTPEEDETIIRAHARFGNKWATIARLLNGRTDNAIKNHWNSTLKRKSSSLSADEGNELADQIFQNEQPPLKRSVSAGSAMPVSGFHFSPGSPSGSDSDSSLHVTSSSQSHLFKPVARAGGVFPPPSIDTSSPSDDPPTSLSLSLPGVDSVEFSNRSVESTQSKNPFQMLPVAMQIPPPPPPPPQAAAVPFQRALNQETAGEQQDKVFVPFSQELLGVMQEMIKAEVRNYMVGIEQQQFQQQQQQQRYQQQQQFQQQNHQMPSGIGLGLCMQQATDGFRNTGANRMGVSKID

>CaMYB44

MDRVKGPWSPEEDELLQQLVQKHGPRNWSLISKSIPGRSGKSCRLRWCNQSSPQVEHRPLTPEEDDTIIRPHARFGNKWATIARLLNGRTDNAIKNHWNSTLKRKCCSLSADEGRELADQIFENHQPPLKRSVSAGSAMPVSGFHFSPGSPAGSDSDSSLHVTSSSQSHVFKPVAKTGGIFPPPSIDISSPSIDPPTSLSLSLPGADLAELSSRSAESTQSKNPFQLSLPPPVQVPPPPPPPPPPPQPQPVTAVPFEQQDKIFVPFSQELLRVMQEMIKTEVRNYMMGIEQKQQHPQQQHYQQQQQFQQQTHQLPSGLGLGLCMQQASDGYRDRAANRMGISKFD

>VvMYB44

MAATRKDVDRIKGPWSPEEDDALQKLVQKHGPRNWSLISKSIPGRSGKSCRLRWCNQLSPQVEHRAFTSEEDDTIMRAHARFGNKWATIARLLSGRTDNAIKNHWNSTLKRKCSAITEDGSFGGDYPPHPLKRSVSAGAAAPVSGLYLSPSSPCGSDVSDSSLPVVSSSHVYRPVARTGGIIPPETTSSSNDPPTSLSLSLPGVDSCEVSNRAPEPNHAPPANPIQMIPAMAPLQQIPMHQHNQPATVPATVLSQGEKPFIPFSAELLAVMQEMIRKEVRNYMAGLEQNGVCLQADGIRNAAVKRIGISKIE

>MdMYB6

MAVSRKDMDRIKGPWSPEEDDSLQRLVQKHGPRNWSLISKSIPGRSGKSCRLRWCNQLSPQVEHRAFTPEEDDMIIRAHARFGNKWATIARLLNGRTDNAIKNHWNSTLKRKCSDGGGVDLNGGYDGHFLRDHEQPPLKRSVSAGSGVPVSTGLYMSPGSPSGSDASDSSAQVMSLSDCHVYRPLARTGGVLPPAETTSSSNNNSSNSEKNDPPTSLSLSLPGVDSEEVSNQVAPVTESTQAPAPAPVSTTSVAVQNIPVELLAVMQGMIRKEVRNYMAGLEQSGVCFQQAAAGNEGFWNVGVKRIGFSRIE

**The sequencing results confirmed the successful knockout of the** ***NtMYB44* gene.**

Note:The wild-type sequence represents the original target site sequence of the *NtMYB44* gene.

WT Target Sequence (5'→3')：ACCTTCATCAGCACCTAAGAGAGG

Control plant: refers to the unedited wild-type tobacco.

t1 CCGGTAAATCTTGCCGGTTA AGG

t2 CAGCAGCTCGTTCATAAACA TGG

t3 ACTATAGCCCGACTTCTTAA TGG

t4 ACCTTCATCAGCACTAAGAG AGG

SmMYB44

ATGGCGAGTACCTGTAACAGTTCCAAGAGAGATATGGATCGGGTTAAAGGTCCATGGAGCCCCGAAGAGGATGAGCTTTTACAGCAGCTTGTTCAGAAACATGGCCCTAGAAATTGGTCCCTTATAAGCAAATCTATCCCGGGGAGATCCGGTAAATCTTGCCGGTTGCGGTGGTGTAATCAGTTATCTCCTCAGGTAGAGCATAGGGCTTTTACTCCCGAAGAAGATGAGACTATTATTCGGGCTCATGCCCGATTCGGGAACAAATGGGCCACCATAGCCCGACTTCTTAATGGCCGGACTGATAACGCTATTAAGAACCACTGGAATTCTACCTTGAAGAGGAAGTGCTCTTCGCTTAGTGCCGATGAAGGTAACGAACTCGCCGATCAAATTTTTGAGAATCAGCAACCGCCGTTGAAGAGATCCGTCAGTGCCGGATCCGCTATGCCGGTTTCGGGTTTTCATTTCAGTCCGGGTAGTCCGTCGGGTTCGGATAGTGATTCGAGTCTTCATGTTACGTCATCGTCTCAATCTCATGTGTTTAAGCCTGTTGCTAGAACCGGCGGCATCTTTCCGCCGTCGATTGATATTTCATCTCCGCTTGTTGATCCTCCCACCTCTCTCAGCCTTTCGCTTCCCGGTGTTGACTCAGCCGAGTGCTCGAACCGTTCGTCCGAATCGACTCAGTCGAAAAATCCTTTCCAGTTGATGCTTCCGGCGATGCAGATTCCTCCACCACCACCACCGCCGCCGCCTCCGTCTCAGTCTACGGCGGTGCCGTTCGAACGAGTTTCTGCAATCCAACAGTCTCATCAGAACCCTGCCGGAGTCAGCGAACAGCCAGACAAGGTTTTCGTGCCGTTTAGCAAGGAGTTGTTAGGTGTGATGCAGGAGATGATAAAGACGGAGGTGAGGAATTACATGATTGGAATTGAACAAAGGCATCAGCAACAGCAGCAGCAGCAGCATTATCAACAACAGCAGCAGTTTCAGCAACCAAGTCATAAACAGCCAAGTGGTTTAGGGCTTGGCTTGTGTATGCAACAAGCTTCTGATGGATTCAGGGATAGGGCTGCTAATCGCATGGGAATAAGCAAATTCGATTAG

LOC107767835-基因组（NtMYB44）

TCTGCCCTCTCCCCACTTTTTTTTTTTTTTTTTTTTTTTTTTTTTTTTTTTTAATTACTATATTCTCTTTTACTAAATATTTTAATCTAAATCCTTTACCCGTTTCAGTATTCACACTAAAATCTAGGGTCCGATAATAGAGATTACTCAAAGTTTCATTAAATAAAGAGAAAGTAGATGAGAAGGAAGGTCGTAGGAAAGTGACTAGTGTTTTTGGGAAGCGTGTATGAGAGCGTGGGTTACCTGATCCGTACATGTATACTTTATGCGGAAAGGACGGTTTCATGCACATCTTTATAGGAACAAAATTTTTCCTACCCAACATATGTGCTAATTTTTAGTTAAGTGATAAATTAATACTCTACAAAATTAACAAAATATATGCAAATTATCTAGAATTTAAATTTTATTCTATATTTATGTAGTGTTATAGTATCGATTTATCTGGCGCAATACTTTTGACATAGAGCATAATCTAATATAAAAATTTAGTAGAATTATTATACTTAATAGGTCTGAACTCATAATTTTAAAATTATAATAAGTTCAATATTAAAAACTTTAAAAAGGTTGAAAGGAGTGATTTGGTATGCAAGTGGGATAAAGTGGGAAATTAAATTTATGTTGTATTTAATTGAGGGAATAAATACATCGCAAGTAAATTTATACAATAAAACTGCAGATAGTATAAATTTAATACCAAAGTTAATCCTAAATATTCATCTTATTCCATCAAGTTTGAGATTATTTTATCAAATTTCAAAGTAAAATAAATTAATCAAGAAATTATAATTTTAGAATTATCATCCCAAAATAACACACAAATAAAATATACACCGAATTTATTATTATTATTGTTATTATTATTATTATTATCGCAAAATAATTTAGTTTACATATCAAACGATGATCCTAAATTCCTTCTGTTTATATTAAAAAAGAATATTAATGCGTTTATTGCTTGATAGATGAGTCAATTGAAATAAATATTTGCCTGTATTAAAAAGTGGCGGTGACCGATTGACGGGTAGCAGCAGGTTTTCATGGGGATAAGAGTTGGGACAAAGGCCGTTGGAGTTGCAATTAGAATTAGAATGAAAGGAGGGATATGTATATTCAAGGAAGTTGAAGGGGTTTAAGAATAAACAAAATAAGAAACAGGCTGGACTGACAGGATTGTAGTAATGATTCGCCTTCCGATTCGGCGACCAAAGTTGTCGTTTAGTGCCAGCTCAGCACGTCCGTGCGGCCGTTTCATTTTCAGGAGGGGTGTTGGGTTGAAATATAAAAAATAAAAAAATAAAAGGAGTGGGTAGATAAATATCCCATCCCCATAAAAAAAAAAAGAAAACGTTATATGCTCTCCAGGCTGTCAACTGCTTGCTTTCAGTCAAAGCTGATGGCCTCGCTCTCTTTCTATTTTTTCTTTGTCTTTTCATCAATCATCTCCTCAACCGTTACCCTAATCTTCTCTCTCTCTCTCTTCCACTCACACCCATACTTATTTTAATTAAAAAATAAATAAATGTATCATGATTTCAGTTACAAGTCACTTGCTTTATTTCTCTTTAATAAAGTCTTAAATCTACAGAGGATTTTCATCATTCTTTAACTGATTTACTGTAGCATGAATAATGAGTTGAGATTAACTCATTCTTTAACTGTGATACATTTACCAGCTCATTCAATGCAATAAACATTTAGGGTATTAGTAGGAAGTATGGTGGAATAATTAAAAAAAATTCTACGTACAAAAAGATAGAGAGAGTGTTACTAATTTAATCGACCTATTCAGTTCAAATTAATGGATCAATTCTAAATATATATCTATCTTAAGGGGAAAAATGAACATTTGAAGTTAAAATTTGTTTTCACTATTTTGCTTCTCCTACATAGTGGAACCCAAATTAACAACCTCTGACTCATTTCAACTTTTGTTTGTTTCCACTAGGATCAAACAAATCTCGTTGGGAAAAAAAAAAGAAGAGTAATGTAGACGCAGCTTATTTATTTATTTATATATTTATTTATAGAAAAAGAGCCCTAAAACTGAAACAGGAGCCATCTGTTATTGTGTAAATCAGTGTAATCTTTCTTTATGACGCGTGTCCCTGTTTACAGCTGTATTCCATCTCAACCGCTTTCCCTATCCCTCCTTTTTTTTAAAAATCAATCGGTGCCCATAACGCTTACAACTCTTCCCCACTTACCGGCTTTACCGGTCATCCCTTTTCCCTTTTATACCCTCTTCTCCCCATATATAAATCTCCGCTTCTTCACGTTTCCTCTTCCTAATCCTAAAACACAAACCACACACAAAAATGGCCAATGGCAGTAACTGTTCTAAGAAAGATATGGATCGGGTTAAAGGTCCATGGAGCCCCGAAGAAGACGAGCTTCTACAGCAGCTCGTTCATAAACATGGACCACGAAATTGGTCTCTTATTAGCAAATCCATACCTGGAAGATCCGGTAAATCTTGCCGGTTAAGGTGGTGCAATCAGTTATCTCCTCAAGTAGAGCATCGCGCTTTTACTCCCGAAGAAGATGAGACCATTATTCGGGCTCATGCCCGATTTGGGAATAAATGGGCCACTATAGCCCGACTTCTTAATGGACGGACCGATAACGCCATTAAGAACCACTGGAACTCTACCTTGAAGAGGAAGTCCTCCTCTCTTAGTGCTGATGAAGGTAACGAACTCGCCGATCAAATTTTTCAAAATGAACAGCCGCCGTTAAAGAGATCCGTTAGTGCCGGATCCGCTATGCCGGTGTCGGGTTTCCATTTCAGTCCCGGTAGCCCGTCGGGTTCGGACAGTGATTCGAGCCTTCATGTCACCTCATCGTCTCAATCTCACTTATTCAAGCCTGTCGCTAGAGCTGGCGGTGTGTTTCCGCCGCCGTCTATTGACACGTCATCTCCTTCCGATGATCCGCCGACTTCCCTCAGCCTTTCGCTTCCCGGAGTTGACTTGGCCGAGTTTTCTAATCGTTCGGCTGAGTCAACTCAGTCGAAGAATCCCTTCCAGATGCTTCCTGTTGCTATGCAGATTCCGCCACCTCAGGCGGCGGCGGTGCCATTTCAATGTGCTCCGTTAAACCAGGAAGCTGCAGGAGGAGAGCAAGATAAGGTGTTTGTGCCTTTTAGTCAGGAGTTGTTGGGAGTGATGCAGGAGATGATTAAAGCGGAGGTGAGGAACTATATGGTGGGAGTTGAACAGCAACAATTTCAGCAACAACAACAGCAGCAATGTTATCAACGACAACAGCAGTTTCAGCAACAAAATCATCAAATGCCAAGTGGAATAGGTCTAGGTTTGTGTATGCAACAAGCTACTGATGGATTCAGGAATACAGGTCCTAATCGCATGGGAGTAAGCAAAATCGATTAGAGAGAGGGAGGTGGGGAAAGAAACAGCCTTTGTTTCATTTCTCTCTATGTTTAGGGGGGCACATGTTGCCTTTGAGCTTCAACTGTGTGGGATCGTCAAAAAAAGGAAGTACTTTTTGGACTGTTTGTACAGAGAGTGGGAGGGAGAGTATAAAGCCAAGTTCCACTGCCAGAAATATGCTAAGGCTGGAACAAATAAGAAAAGAAAAGCCAAAAAGATTAAAAAAGACGTTTTGTTAATTTGTATTCAGGCAGATTTAGAAAACAAGAACAAAACTAGGTATTGAGAGACCCTACCGCCAAAGAAATGAAAAATGAAATCTGGTGGAAGGATTTGAATGAAGCAAAGTTCTTAACTAAGTTATTTATCTTTCCTTTTCAAATTTAAACGCTGATGACCAGATGAGTTTGAAAATCGCAGGGAACTGAAAGAAAGAATTGAATTGTGGACAATACAGTATGAGTAATTCCATAGTGAAGCTGAAAACATACAGCAGCAATTAGAAAGGTCACAAATTATAAGGATTGCATCAAGGTATTTGGGAACTTGTCGCCAATGTGGAGGTTGCAGTGTTTGAAAATGAACTAAACCTTAATTATTAGCCGAAAGTGATAAGCACAAGGAGAAGGTAGAAAACTAATTTAACCAAAAAAACCTTTTCGAATATGATTCAGATCAAAAATAATCTATAGCCATATTCCATTACTTTCTCCTCTTCATATGAAACTGGTTGCAAACAAACTTACAAAACGTAGAAGATTGGAGAGAGTTGCTGTTTTACCTTTTTCCTTTTTAAAGAAATCTAGTCGGCCTCTACCTTTCACATATGCCCTTATACTCAGAATAGATATGTATAAGCTGGTCGTAAAAGTGGTTTGTCAGTTTTGCATTCTCAAATTATACAGATTCCATCATTTTTGTGAGAGTTCAATTGATCTTTTTTCCATCTTTAGAGCCGCGTGTAAACACATGAAAAACTGTTCGAGCGCATCATCATAACTGAAATTGAACGTTTGCACGTTTGACCAAGCCATTAAGGTAAAGATAAGACCTCCTTTTTCCAATAGGAGACTTGTACAAATGTGGTACAAGACAACCAACTTTATGATTAATGGTTCCCTAACGTCCTGCATTAAAATTAGATGCTTTGCCGCTTACATCTTTACAGTAACATTTCAATACCGCCTAATATCATGCAAAGTACATAAACATGGAAAAGAATATACATAGGGCTGATTTAGCCTCACGTGTACACCTGACAATCTAGTTGGTGCGGCTCAACATGTACAGTGCAGTATTCTTCACTTTCTGACAACAAAGTTTTCGTCACTCCATCTCAATTGGCATGTGAACAGCTACAGTGCAGGAGGTCAATACTCACAAATGGGCTGCTTGAATGGTGCTAGGTAGGGTGCCATGCCTCAAGAACATAATTAGAGAATGCAATTTGTCAATCATTTGTGAAACATCCTCCCTCCATGACCATGTTGGAGGATTTAGAGCACGAACAGAATTGAGACAAACAAGAAGCGTGCCACCTTCATGCAAGAGGACCTGCATGTTGACATACCAGCTCAAATAAATTTACTAAGATAGCCACCAAGCTAGGGCAAAAATGCAGTTTCTCATTTTCCGTAGTTGCAAGTCCCCAACAGAATGTAAATCTATCTATCTTCCAAGAATAACAAGAATTGGACCACTTAGAAATAAGAAAATTTCACAAAGGTTAACTTGTGTTTCCTACTAGGTTATATTGTTGGACCTAATAGCTGCACAAAGACTAACAGCAGCAAGCTGGACAGATATACAGATTGATTGAAACAAGATGGATAAACTTAAGGTGAGAAATATCTGGAGTAGAGTTATACCGTCAACCACAGTGGGAGGACTCCCAATACTGATGTAAGAGAGGCCAAAAGAATGCTAGACAAAGCAAGTGCTACATTTTGCTTGATCTGCAAGTATTGGGGTGAGCATCAGCTGCATATAGAAAGACGAGTTGATTACAAAGGAAAAGGAAATACCAAGGATGTTGTTTGACGAGATTTCGCAACACAAAATGGGACACCGGAAATATTGTCTTGTAACAACAGTACATCGGCAACAGCTATGGCAGCCGCACTAGCTCGTTCAGCCAGCACAATACCAACAGTAGCTGCAGCAAGTGCTGGGGCATCATTGATGCCATCACCTACCATTATAAGGCCGCCTGCTCAGAAGTATAGTAATTGGTCAGGTAACAATTAGCACAATAAGGATACATGAATTATCGTGAAAAGTTATAGTTGATTAGCATCTACCCAAGTTTGCACCCCATACATACACATTCCTGGGGCCCTAAAGTGGAGAGAAATGAGAATCATGACTAAGGTTTACCCTTTTTCGACTTCTCCTTGTTACCATGAACAGCAAAATAACTTCGGGTGTATGCTTTATTCCATCCAGTTTCACCTATAAGGTCCTCTGTTCTAAGTGGGATCTCCAATTTATTTAACTTTTATAGTCTATAGACATGAGTGGGTCCCCACAGAAGCATATGTAAACTTGTTTATCTTGATGGTTTAAAGTTAAAACTTTAATGCTTTAGCATTGGTTAAATTAATTTGTTATTGACTATTTAGAAAACGTCAACATAAAGACGCTAAATAAATATCAAAATTCCGCCATTAGTTTGATAAGTTGAATCTTTTAGGTCGCAGTCCAATGATTTGTTGCATGAAGGTAGCTTCTTCTCCTCTTTCGTTTGTATGTCTTTTTTAACTTAATACTTTATATCATTTCTTATTTGTGCTAATTTCTGAGTTGACTGACTCAAAGGTCTAAAAACACAGTTCTCATCTCTAAACCAATATCCAAAGGAATTCTCAAGGAGTGAAACAAAACTTGAAGTTCCAGAAAGTACGGAACAAAAACCAAGGAGCAATTTACAAATCTGCTTTAGCTAGCTGAAGCTCAGGTGAGTAGCAGGACAAGACATG

2

CGACGGTCTCGGGATGAATGGAACCCGACACCGGCATAGCGGATCCGGCACTAACGGATCTCTTTAACGGCGGCTGTTCATTTTGAAAAATTTGATCGGCGAGTTCGTTACCTTCATCAGCACTGAGAGGAGGACTTCCTCTTCAAGGTAGAGTTCCAGTGGTTCTTAATGGCGTTATCGGTCCGTCCATTAAGAAGTCGGGCTATAGTGGCCCATTTATTCCCAAATCGGGCATGAGCCCGAATAATGGTCTCATCTTCTTCGGGAGTAAAAGCGCGATGCTCTACTTGAGGAGATAACTGATTGCACCACCTTAACCGGCAAGATTTACCGGATCTTCCAGGTATGGATTTGCTAATAAGAGACCAATTTCGTGGTCCATGTTTATGAACGAGCTGCTGTAGAAGCTCGTCTTCTTCGGGGCTCCATGGACCTTTAACCCGATCCATATCTTTCTTAGAACAGTTACTGCCATTGGCCATTTTTGTGTGTGGTTTGTGTTTTAGGATTAGGAAGAGGAAACGTGAAGAAGCGGAGATTTATATATGGGGAGAAGAGGGTATAAAAGGGAAAAGGGATGACCGGTAAAGCCGGTAAGTGGGGAAGAGTTGTAAGCGTTATGGGCACCGATTGATTTTTAAAAAAAAGGAGGGATAGGGAAAGCGGTTGAGATGGAATACAGCTGTAAACAGGGACACGCGTCATAAAGAAAGATTACACTGATTTACACAATAACAGATGGCTCCTGTTTCAGTTTTAGGGCTCTTTTTCTATAAATAAATATATAAATAAATAAATAAGCTGCGTCTACATTACTCTTCTTTTTTTTTTCCCAACGAGATTTGTTTGATCCTAGTGGAAACAAACAAAAGTTGAAATGAGTCAGACGTTTTAATTTTGGGGTACGTAT

3

CCATGGGCTCGGGACTGAATGGAACCCGACACCGGCATAGCGGATCCGGCACTAACGGATCTCTTTAACGGCGGCTGTTCATTTTGAAAAATTTGATCGGCGAGTTCGTTACCTTCATCAGCACTAAAACATGGACCTATAATGGCCCCTTTTTTCCCCAATCCGGCATGAGCACGAATAAGGGTCTCTTCCTCGTCAGGGGGGAGCACTCGATGCTCTCCTCGAGGAGATCACTGATCGCTCCCTCTTAACCAGCATGATATACCGGATCTTCCTCGTGCGGAATTTCTGAATAAATGGGCCTTTCTAGCCCCACTTTTTTAATGGACCGGCTGATAACGCCCTTCTTATTCACTGCACCTCGGACCTTAAAACGAATCCCTCTCCTCCTTATAAACGATCTTGTGTTTAACCTTTTCTTCTTGTGGGTTCCGTGGACCGATTACGAAAACGATATCTTTCTTAGCGGATTACTGCCATTGGGATTTTTGGTGTGGTTGGAAAAGGGATGACCGGAAGAGGAAACGTGAGGGGAAGAGTATTTATATATGGGGACACCAAGGGATTTTTAGGAAAAAGGATGAACAGGGAAACCGGTTGATATGGAATACTTCTGTACGTTAGGACCCCCGATTGATTTATAGATTACACTGATTAACAGGAAAGCGATGGCTCCTGTTACAGCTGTAAGGCTCTTTTTCTATCATAAATATATATTACACTGATTAACCTGCAACTACATGGCTCCTGTTTCTTTTTTCCGGCTCATTTTCTATAAATACATATAGAAATCAATAAATAATCTACGTGAACATACTCTTCTTTTTTTGTGTACAACGTATTTTTCGTCTGTGACTATTAGTGAAACAAACAAAAGTTGAAATGAGTCAGAGGTTGTTAATTTTGGGTACA

6

CACCGGGCTCGGGACTGAATGGAACCCGACACCGGCATAGCGGATCCGGCACTAACGGATCTCTTTAACGGCGGCTGTTCATTTTGAAAAATTTGATCGGCGAGTTCGTTACCTTCATCAGCACTAAAGAGAGGAGGACTTCCTCTTCAAGGTAGAGTTCCAGTGGTTCTTAATGGCGTTATCGGTCCGTCCATTAAGAAGTCGGGCTATAGTGGCCCATTTATTCCCAAATCGGGCATGAGCCCGAATAATGGTCTCATCTTCTTCGGGAGTAAAAGCGCGATGCTCTACTTGAGGAGATAACTGATTGCACCACCTTAACCGGCAAGATTTACCGGATCTTCCAGGTATGGATTTGCTAATAAGAGACCAATTTCGTGGTCCATGTTTATGAACGAGCTGCTGTAGAAGCTCGTCTTCTTCGGGGCTCCATGGACCTTTAACCCGATCCATATCTTTCTTAGAACAGTTACTGCCATTGGCCATTTTTGTGTGTGGTTTGTGTTTTAGGATTAGGAAGAGGAAACGTGAAGAAGCGGAGATTTATATATGGGGAGAAGAGGGTATAAAAGGGAAAAGGGATGACCGGTAAAGCCGGTAAGTGGGGAAGAGTTGTAAGCGTTATGGGCACCGATTGATTTTTAAAAAAAAGGAGGGATAGGGAAAGCGGTTGAGATGGAATACAGCTGTAAACAGGGACACGCGTCATAAAGAAAGATTACACTGATTTACACAATAACAGATGGCTCCTGTTTCAGTTTTAGGGCTCTTTTTCTATAAATAAATATATAAATAAATAAATAAGCTGCGTCTACATTACTCTTCTTTTTTTTTTCCCAACGAGATTTGTTTGATCCTAGTGGAAACAAACAAAAGTTGAAATGAGTCAAAGGTGTTAAATTTTGGGGAAGGGGTTCCCC

11

AACGGGCTCGGGACTGAATGGAACCCGACACCGGCATAGCGGATCCGGCACTAACGGATCTCTTTAACGGCGGCTGTTCATTTTGAAAAATTTGATCGGCGAGTTCGTTACCTTCATCAGCACAAAAAGAAGAATTTCTCCTCCAAGGAAAATTCCCATGGGTCCTTATGGCCTTATCCGGCCCTCCCTTAAAAAATCCGGCTTAAATGGCCCCTTTTTTTCCCAATCCGGCCTTAACCCCAATTATTGTCCCCTCCTCCTCCGGAATAAAAACCCCAATCTCCTCTTTAAGAAAATACTTGTTTCCCCCCCTTTACCCGCCAAAATTTCCCGAACCTTCCAGGAATGAATTTCCTATTAAAAAACCATTTCCTGGGCCATTGTTAATAACCAACCTCCGGAAAAACCCCTCCTCCTCCGGGCTCCCTGGAACTTTAACCCCAACCCTATCCTTCCTAAAACCATTACCGGCCTTTGCCCTTTTTTGGGGGGGGTTGGGGTTTAAGGATAAGAAAAAGAAACCTTAAAAAACCGAAAATTATTTTTTGGGAAAAAAAGGGAATAAAAGGAAAAAGGAATAACGGGAAAACCCGGAAATGGGGAAAAATTTGAAACCTTATTGGCCACCAATTATTTTTAAAAAAAAGGAGGAAAGGAAAACCGGTGAAAATGAATCCACTGGAAACAGGACCCCCTCATAAGAAAAATACCCTGATTACCCATAACAAAGGCTCCTGTTCAGTTTAGGCTCTTTTCTATAATAATATATAATAATAATAGCTGCGCCACATACTCTCTTTTTTTTCCCACGAAATTGTTGATCTATGGAACAACAAAGTGAATGATCGAGTGTAATTTTGGGAAAAATT

33

CGGGACTGAAATGGAACCCGACACCGGCATAGCGGATCCGGCACTAACGGATCTCTTTAACGGCGGCTGTTCATTTTGAAAAATTTGATCGGCGAGTTCGTTACCTTCATCAGCACTAAAGAGAGGAGGACTTCCTCTTCAAGGTAGAGTTCCAGTGGTTCTTAATGGCGTTATCGGTCCGTCCATTAAGAAGTCGGGCTATAGTGGCCCATTTATTCCCAAATCGGGCATGAGCCCGAATAATGGTCTCATCTTCTTCGGGAGTAAAAGCGCGATGCTCTACTTGAGGAGATAACTGATTGCACCACCTTAACCGGCAAGATTTACCGGATCTTCCAGGTATGGATTTGCTAATAAGAGACCAATTTCGTGGTCCATGTTTATGAACGAGCTGCTGTAGAAGCTCGTCTTCTTCGGGGCTCCATGGACCTTTAACCCGATCCATATCTTTCTTAGAACAGTTACTGCCATTGGCCATTTTTGTGTGTGGTTTGTGTTTTAGGATTAGGAAGAGGAAACGTGAAGAAGCGGAGATTTATATATGGGGAGAAGAGGGTATAAAAGGGAAAAGGGATGACCGGTAAAGCCGGTAAGTGGGGAAGAGTTGTAAGCGTTATGGGCACCGATTGATTTTTAAAAAAAAGGAGGGATAGGGAAAGCGGTTGAGATGGAATACAGCTGTAAACAGGGACACGCGTCATAAAGAAAGATTACACTGATTTACACAATAACAGATGGCTCCTGTTTCAGTTTTAGGGCTCTTTTTCTATAAATAAATATATAAATAAATAAATAAGCTGCGTCTACATTACTCTTCTTTTTTTTTCCCAACGAGATTTGTTTGATCCTAGTGGAAACAAACAAAAGTTGAAATGAGTCAGAGGTTGTTAATTTGGGGTAATCCTCCTTTCT

34

CGGGACTGAATGGAACCCGACACCGGCATAGCGGATCCGGCACTAACGGATCTCTTTAACGGCGGCTGTTCATTTTGAAAAATTTGATCGGCGAGTTCGTTACCTTCATCAGCACTAAAGAGAGGAGGACTTCCTCTTCAAGGTAGAGTTCCAGTGGTTCTTAATGGCGTTATCGGTCCGTCCATTAAGAAGTCGGGCTATAGTGGCCCATTTATTCCCAAATCGGGCATGAGCCCGAATAATGGTCTCATCTTCTTCGGGAGTAAAAGCGCGATGCTCTACTTGAGGAGATAACTGATTGCACCACCTTAACCGGCAAGATTTACCGGATCTTCCAGGTATGGATTTGCTAATAAGAGACCAATTTCGTGGTCCATGTTTATGAACGAGCTGCTGTAGAAGCTCGTCTTCTTCGGGGCTCCATGGACCTTTAACCCGATCCATATCTTTCTTAGAACAGTTACTGCCATTGGCCATTTTTGTGTGTGGTTTGTGTTTTAGGATTAGGAAGAGGAAACGTGAAGAAGCGGAGATTTATATATGGGGAGAAGAGGGTATAAAAGGGAAAAGGGATGACCGGTAAAGCCGGTAAGTGGGGAAGAGTTGTAAGCGTTATGGGCACCGATTGATTTTTAAAAAAAAGGAGGGATAGGGAAAGCGGTTGAGATGGAATACAGCTGTAAACAGGGACACGCGTCATAAAGAAAGATTACACTGATTTACACAATAACAGATGGCTCCTGTTTCAGTTTTAGGGCTCTTTTTCTATAAATAAATATATAAATAAATAAATAAGCTGCGTCTACATTACTCTTCTTTTTTTTTCCCAACGAGATTTGTTTGATCCTAG

37

AAGGGCAACGGGACTGAATGGAACCCGACACCGGCATAGCGGATCCGGCACTAACGGATCTCTTTAACGGCGGCTGTTCATTTTGAAAAATTTGATCGGCGAGTTCGTTACCTTCATCAGCACTAAAAAAAGAAGAATTCCTCCTTCAAGGAAAATTCCCATGGGTCCTTATGGCCTTTTTCGGCCCTCCCTTTAAAAATCCGGCTAATATGGGCCCTTTATTCCCCAATCCGGCCTTAACCCCAATTATTGGCCCCTCCTCCTTCGGAATAAAAACCCCAATGCCCTACTTAAGAAAATACTGATTTGCCCCCCCTTACCCGCCAAAATTTCCCGAACCTTCCAGGATTGAATTTCCTATTAAAAAACCATTTCCTGGGCCCTTGTTATTAACCAACCTCCTGAAAAACCCCTCCTCCTTCGGGCTCCCTGGAACCTTTACCCCAACCCTAACCTTCCTAAAAACAGTTCCGGCCTTTGCCCTTTTTGGGGGGGGGTTGGGGTTTAAGAATAAGGAAAAGAAACCTTAAAAAACCGAAAATTATATATGGGAAAAAAGGGATAAAAGGAAAAAGGAAAACCGGAAACCCGGAATGGGGAAAAATTGTACCGTTTGGCCCCAATGATTTTTTAAAAAAAGGAGGATAGGAAACCGGTAAAAGAATCCACTGTAACAGGAACCCCCCTAAAAAAATACCCGATTACCCATACAAATGCTCTGTTCATTTAGGCTCTTTTCATAATAATATTAATAATAATAGCTGCTCTCTTATCTCTTTTTTTCCACAAATTGTTGACCTAGGAACAACAAAGTGAAGACAGAGTGTAATTGGTATCTTTACC

43

GGCCACGGGACTGAATGGAACCCGACACCGGCATAGCGGATCCGGCACTAACGGATCTCTTTAACGGCGGCTGTTCATTTTGAAAAATTTGATCGGCGAGTTCGTTACCTTCATCAGCACTAAGAGAGGAGGACTTCCTCTTCAAGGTAGAGTTCCAGTGGTTCTTAATGGCGTTATCGGTCCGTCCATTAAGAAGTCGGGCTATAGTGGCCCATTTATTCCCAAATCGGGCATGAGCCCGAATAATGGTCTCATCTTCTTCGGGAGTAAAAGCGCGATGCTCTACTTGAGGAGATAACTGATTGCACCACCTTAACCGGCAAGATTTACCGGATCTTCCAGGTATGGATTTGCTAATAAGAGACCAATTTCGTGGTCCATGTTTATGAACGAGCTGCTGTAGAAGCTCGTCTTCTTCGGGGCTCCATGGACCTTTAACCCGATCCATATCTTTCTTAGAACAGTTACTGCCATTGGCCATTTTTGTGTGTGGTTTGTGTTTTAGGATTAGGAAGAGGAAACGTGAAGAAGCGGAGATTTATATATGGGGAGAAGAGGGTATAAAAGGGAAAAGGGATGACCGGTAAAGCCGGTAAGTGGGGAAGAGTTGTAAGCGTTATGGGCACCGATTGATTTTTAAAAAAAAGGAGGGATAGGGAAAGCGGTTGAGATGGAATACAGCTGTAAACAGGGACACGCGTCATAAAGAAAGATTACACTGATTTACACAATAACAGATGGCTCCTGTTTCAGTTTTAGGGCTCTTTTTCTATAAATAAATATATAAATAAATAAATAAGCTGCGTCTACATTACTCTTCTTTTTTTTTTCCCAACGAGATTTGTTTGATCCTAGTGGAAACAAACAAAAGTTGAAATGAGTCAGAGGTGTTTATTTTGGGGTAAATGGGGGG

44

TCGGGATTTGGCGGCTCGGGATGAATGGAACCCGACACCGGCATAGCGGATCCGGCACTAACGGATCTCTTTAACGGCGGCTGTTCATTTTGAAAAATTTGATCGGCGAGTTCGTTACCTTCATCAGCACTGAAAAGAGGACGACTTCTTCTTCGTAGAATACCTCCGGTGCTTCTTGGCGGCATCATCCCGCCCTTTATTAAGACGGGCGGCTATGGCCCCTTTATTCTTCCCTCGGGCATGATGACCAATAATGGTGGCCTCTTCTTCTTGAGGAGTAACGCGATGATCTACTTGTTGAGGAAACTGATTGCTGCACCTTATCCGGCGGCATTATTCGGATCATCTTCGTAGGGATGAGCTGCTAATAGAACACTTTCTTGGTCCATGATGTTTAACAACCTGCTGCTGAAGATCGTCTTCTTCTTCGCTGCTTGGACCACCATCCCGATCATCCTCATCCTTCTTACAACTACTGCTGTTGTTGATCATTGTTTGTGTGGGTGTGTATGATGATGAAGAGGAAGCGTCGTGAAGCGGAGATTATTATATGATGAGAGAGGATGGAATAGGGAGATAGATGATCACCGGTGACGGTAGGAGTGAGAGGAGTATCGCATGTCGCGATTGTGATTACAAAGGCGAAGATAGGGAGCTGATATGGAGTATGCTGCTGTCACAAGGACAGCATCAGAGATTACTCTGATATCCACAATAGATGACGGCTGCGCACATATGGCGCATTACATTAATTATAATTAATTAATTATCTGCGCTCTCACTCTCTCTTTCCGAGATGTGTAGTATAGACACCACAGCTCGTGTGATAAGTGATATGGATA

45

GGGCTCGGGACTGAATGGAACCCGACACCGGCATAGCGGATCCGGCACTAACGGATCTCTTTAACGGCGGCTGTTCATTTTGAAAAATTTGATCGGCGAGTTCGTTACCTTCATCAGCACTAAGAGAGGAGGACTTCCTCTTCAAGGTAGAGTTCCAGTGGTTCTTAATGGCGTTATCGGTCCGTCCATTAAGAAGTCGGGCTATAGTGGCCCATTTATTCCCAAATCGGGCATGAGCCCGAATAATGGTCTCATCTTCTTCGGGAGTAAAAGCGCGATGCTCTACTTGAGGAGATAACTGATTGCACCACCTTAACCGGCAAGATTTACCGGATCTTCCAGGTATGGATTTGCTAATAAGAGACCAATTTCGTGGTCCATGTTTATGAACGAGCTGCTGTAGAAGCTCGTCTTCTTCGGGGCTCCATGGACCTTTAACCCGATCCATATCTTTCTTAGAACAGTTACTGCCATTGGCCATTTTTGTGTGTGGTTTGTGTTTTAGGATTAGGAAGAGGAAACGTGAAGAAGCGGAGATTTATATATGGGGAGAAGAGGGTATAAAAGGGAAAAGGGATGACCGGTAAAGCCGGTAAGTGGGGAAGAGTTGTAAGCGTTATGGGCACCGATTGATTTTTAAAAAAAAGGAGGGATAGGGAAAGCGGTTGAGATGGAATACAGCTGTAAACAGGGACACGCGTCATAAAGAAAGATTACACTGATTTACACAATAACAGATGGCTCCTGTTTCAGTTTTAGGGCTCTTTTTCTATAAATAAATATATAAATAAATAAATAAGCTGCGTCTACATTACTCTTCTTTTTTTTTTCCCAACGAGATTTGTTTGATCCTAGTGGAAACAAACAAAAGTTGAAATGAGTCAAAGGTTGTTAATTTGGGGTAAAGAATAAAT

46

GCACGGTCTCGGGACTGAATGGAACCCGACACCGGCATAGCGGATCCGGCACTAACGGATCTCTTTAACGGCGGCTGTTCATTTTGAAAAATTTGATCGGCGAGTTCGTTACCTTCATCAGCACTAGAAGAGGACGACTTCCTCGTCAAGGTACAGTTCCTCTGGTTCTTAATGGCGTTATCGGTCCGTCCATTACGAACTCGGGCTATACTGGCCCTTTTATTCCCAAATCGGGCATGAGCCCTAATAATGGTCTCATCTTCTTCGGGAGTAAAAGCGCCATGCTCTACTTGAGGATATAACTGATTGCACCACCTTAACCAGCAACATTTACCGGATCGTCCGGGTATGGATTTGCTAATAAAATACCAATTTCCTGGTCCATGTTTATGAACCAGCTGCTGTACAACCTCGTCTTCTTCCGGGCTCCCTGGACCTTTAACCCTATCCATATCTTTCTTATAACAGTTACTGCCATTGGCCATTTTTGTGTGTGGTTTGTGTTTTAGGATTAGGAAAAGGAAACGTGAAGAAACGGATATTTATATATGGGGAGAAAAGGGTATAAAAGGGAATAAGGATGACCGGCAAAGCCGGTAAATGGGGAATAGTTGTAATCGTTATGGGCACCTATTGATTTTTAAAGAAGAAGAGGGATAGGGAAAGCGGTTGAGATGGAATACAGCTGTGGACACGCACACTCGTCATAAATAAAGATTACACTGATTTACACAATAACATATGGCTCCTGTTTCAGGTTTATGGCTCTTTTTCTATAAATAAATATATAAATAAATACATAAGCTGCGTCTACCTTACTCTTCTTTTTTTTTCCCAACGAGATTTGCTTGATCCTAACGGAAACAAACAAAAGTGAAACTGAGTCAAGGG

47

CCATGTCTCGGGACTGAATGGAACCCGACACCGGCATAGCGGATCCGGCACTAACGGATCTCTTTAACGGCGGCTGTTCATTTTGAAAAATTTGATCGGCGAGTTCGTTACCTTCATCAGCACTGAAAAGAGGACGACTTCCTCTTCGTAGAATACCTCCGGTGGTTCTTGGCGGCATCATCCCGCCCTTCATTAAGACGGGCGGCTATGGCGGCCCTTTTCCTCCCTCGGGCGTGATGACCCCTAATGGTGGCCTCTTCTTCTTCAGGAGTAACGCGATGATGCTCTACTTGAGGTAATTGATGATTGCACCTTATTCGGCGGCATTAATCGGATCATCTTCGTAGGATGGAGCTGCTAATAGACCACTTTCTTGGTGGATGTTGATTATGAACCTGCTGCAGAAGATCGTCTTCTTCTTCGCTGCTCGGTGGACCTTTCCCATCATTCTCATCCTTCTTACAACTACTGCTGTTGTTGGTCATTGTTTTTTGTGGGTTTTGTATGATGATGAAGAGGAAGCAACGTGAAGAAGAGGTTATTTTTTGATGAGAGAGGATGGATAGGGAGAAGATGATGACGGTGCGGTGTAGTGAGAGGAGTATCGCATGTCGCACTGTGATAAAAAGAGAAGATAAGACGCTGATATGAGTATGCTGTGTCACGACACCGCATCATGAGATTATTCTGATAATCACATAGATGATGGCTCCTCTCATATGACTCTCTCTCATATAGTTATTATTATATCTGCTGCTCACACTCTCTCGTTTCCGACATGTGTAGCTATAGGACACCAAATGTAGTCTCACGGATTTGTGAAA

48

CCAAGGGGCTCGGGGACTGAATGGAACCCGACACCGGCATAGCGGATCCGGCACTAACGGATCTCTTTAACGGCGGCTGTTCATTTTGAAAAATTTGATCGGCGAGTTCGTTACCTTCATCAGCACAAAGGAGGACATCATCTCCTCTGCAAAGTTCAATTGGTTCTTATTGGCGTGGTCTTACCGGCCCTTCCAAAATCAGGCTAGGCTGGCCCGTTTCTTCCCTTCTCGGGCATGACCCCAACTAAAGGACTCGTCTCCTTCTCGAGTAAAATCACGATGCTCTGCTCGAGGAGATGACAGATTGCACCGCCTCACCCTGCCAGATTTAACTGATCTTACCTGCATGGATTTGATTTTCTAATACCAAATTCATGGCCCGTGCTTATGAACTAACTGATGTGCAGTCTCATCTTCTTCTCGGCTCGGTGCACCTTACCCCCAATCCAAATCTTTCTTATAATAGATACTGTACTGGCCCTTTTTTGTGTGGTGTGTGTGTTGTGTGATTAGAATAAGGAAACGTGAAGTGACGAAGATTTGATTATGGGGAGAAGAGAGTATGGTATGGAAGGGGGATGACATGACCAGCAGGTCAGTTGAGTAGAGAAGAGTGCTATACGTGCTGCGCATCGATTTTATTATAAAAGAGGGTAGGAAAGCAGATCAGATGAATACACTCTACTGTGGACAGCCTCACATATAAGATACACTGATTGATTACACATATCGCACGTCTCTGTTCATGCACGCTCTATCTAATTATAATAATAATAGCTGCGCTGACATACACTACTTCTTTTCCTCCAACTAGATTGATCGATCGATCGACAAACTGAATGATCGATGTAGTATTGAGTTAGGTA

**Table S4 Cis-acting regulatory elements analysis of *SmCHS* promoter sequences**

| **Motif name** | **Motif sequence** | **Position** | **+** | **Promoter Sequence Element Function Annotation** |
| --- | --- | --- | --- | --- |
| CGTCA-motif | CGTCA | 620 | + | cis-acting regulatory element involved in the MeJA-responsiveness |
| CGTCA-motif | CGTCA | 1322 | - | cis-acting regulatory element involved in the MeJA-responsiveness |
| CGTCA-motif | CGTCA | 1930 | - | cis-acting regulatory element involved in the MeJA-responsiveness |
| CAT-box | GCCACT | 2085 | - | cis-acting regulatory element related to meristem expression |
| AT1-motif | AATTATTTTTTATT | 2013 | + | part of a light responsive module |
| Unnamed__1 | GAATTTAATTAA | 305 | - | 60K protein binding site |
| I-box | GTATAAGGCC | 665 | - | part of a light responsive element |
| Box 4 | ATTAAT | 37 | + | part of a conserved DNA module involved in light responsiveness |
| Box 4 | ATTAAT | 76 | + | part of a conserved DNA module involved in light responsiveness |
| Box 4 | ATTAAT | 346 | + | part of a conserved DNA module involved in light responsiveness |
| Box 4 | ATTAAT | 554 | + | part of a conserved DNA module involved in light responsiveness |
| Box 4 | ATTAAT | 780 | + | part of a conserved DNA module involved in light responsiveness |
| Box 4 | ATTAAT | 1408 | - | part of a conserved DNA module involved in light responsiveness |
| Box 4 | ATTAAT | 1573 | - | part of a conserved DNA module involved in light responsiveness |
| Box 4 | ATTAAT | 1653 | - | part of a conserved DNA module involved in light responsiveness |
| Box 4 | ATTAAT | 1840 | - | part of a conserved DNA module involved in light responsiveness |
| Box 4 | ATTAAT | 1891 | - | part of a conserved DNA module involved in light responsiveness |
| TGACG-motif | TGACG | 620 | - | cis-acting regulatory element involved in the MeJA-responsiveness |
| TGACG-motif | TGACG | 1322 | + | cis-acting regulatory element involved in the MeJA-responsiveness |
| TGACG-motif | TGACG | 1930 | + | cis-acting regulatory element involved in the MeJA-responsiveness |
| G-Box | CACGTG | 2378 | - | cis-acting regulatory element involved in light responsiveness |
| AT-rich element | ATAGAAATCAA | 2038 | + | binding site of AT-rich DNA binding protein (ATBP-1) |
| G-box | CACGTG | 2378 | - | cis-acting regulatory element involved in light responsiveness |
| O2-site | GATGATGTGG | 1397 | - | cis-acting regulatory element involved in zein metabolism regulation |
| O2-site | GATGA(C/T)(A/G)TG(A/G) | 1491 | - | cis-acting regulatory element involved in zein metabolism regulation |
| MRE | AACCTAA | 2206 | + | MYB binding site involved in light responsiveness |
| ABRE | CACGTG | 2378 | - | cis-acting element involved in the abscisic acid responsiveness |
| ABRE | ACGTG | 2379 | + | cis-acting element involved in the abscisic acid responsiveness |

**Table S5 Cis-acting regulatory elements analysis of *SmF3H* promoter sequences**

| **Motif name** | **Motif sequence** | **Position** | **+** | **Promoter Sequence Element Function Annotation** |
| --- | --- | --- | --- | --- |
| CCAAT-box | CAACGG | 1264 | + | MYBHv1 binding site |
| TATC-box | TATCCCA | 368 | - | cis-acting element involved in gibberellin-responsiveness |
| chs-CMA1a | TTACTTAA | 1160 | + | part of a light responsive element |
| AT1-motif | AATTATTTTTTATT | 789 | - | part of a light responsive module |
| AT-rich element | ATAGAAATCAA | 2373 | + | binding site of AT-rich DNA binding protein (ATBP-1) |
| G-Box | CACGTG | 596 | + | cis-acting regulatory element involved in light responsiveness |
| G-Box | CACGTG | 1770 | - | cis-acting regulatory element involved in light responsiveness |
| G-Box | CACGTG | 2614 | - | cis-acting regulatory element involved in light responsiveness |
| GATA-motif | GATAGGA | 950 | + | part of a light responsive element |
| GATA-motif | AAGGATAAGG | 2675 | - | part of a light responsive element |
| O2-site | GATGACATGG | 47 | + | cis-acting regulatory element involved in zein metabolism regulation |
| O2-site | GTTGACGTGA | 1053 | + | cis-acting regulatory element involved in zein metabolism regulation |
| O2-site | GATGACATGG | 1676 | + | cis-acting regulatory element involved in zein metabolism regulation |
| G-box | CACGTG | 596 | + | cis-acting regulatory element involved in light responsiveness |
| G-box | CACGTG | 1770 | - | cis-acting regulatory element involved in light responsiveness |
| G-box | CACGAC | 2007 | + | cis-acting regulatory element involved in light responsiveness |
| G-box | CACGTG | 2614 | - | cis-acting regulatory element involved in light responsiveness |
| GT1-motif | GGTTAA | 260 | - | light responsive element |
| MRE | AACCTAA | 262 | + | MYB binding site involved in light responsiveness |
| MRE | AACCTAA | 2164 | + | MYB binding site involved in light responsiveness |
| ARE | AAACCA | 2721 | + | cis-acting regulatory element essential for the anaerobic induction |
| MBS | CAACTG | 621 | - | MYB binding site involved in drought-inducibility |
| GA-motif | ATAGATAA | 80 | - | part of a light responsive element |
| GA-motif | ATAGATAA | 1565 | + | part of a light responsive element |
| GA-motif | ATAGATAA | 1580 | - | part of a light responsive element |
| GA-motif | ATAGATAA | 2481 | + | part of a light responsive element |
| TCT-motif | TCTTAC | 2128 | - | part of a light responsive element |
| TCT-motif | TCTTAC | 2322 | + | part of a light responsive element |
| ABRE | CACGTG | 596 | + | cis-acting element involved in the abscisic acid responsiveness |
| ABRE | ACGTG | 597 | + | cis-acting element involved in the abscisic acid responsiveness |
| ABRE | CACGTG | 1770 | - | cis-acting element involved in the abscisic acid responsiveness |
| ABRE | ACGTG | 1771 | + | cis-acting element involved in the abscisic acid responsiveness |
| ABRE | CACGTG | 2614 | - | cis-acting element involved in the abscisic acid responsiveness |
| ABRE | ACGTG | 2615 | + | cis-acting element involved in the abscisic acid responsiveness |
